# An integrative and applicable phylogenetic footprinting framework for *cis*-regulatory motifs identification in prokaryotic genomes

**DOI:** 10.1186/s12864-016-2982-x

**Published:** 2016-08-09

**Authors:** Bingqiang Liu, Hanyuan Zhang, Chuan Zhou, Guojun Li, Anne Fennell, Guanghui Wang, Yu Kang, Qi Liu, Qin Ma

**Affiliations:** 1 School of Mathematics, Shandong University, Jinan, 250100 China; 2 Systems Biology and Biomedical Informatics (SBBI) Laboratory University of Nebraska-Lincoln, Lincoln, NE 68588-0115 USA; 3 Department of Agronomy, Horticulture, and Plant Science, South Dakota State University, Brookings, SD 57007 USA; 4 BioSNTR, Brookings, SD USA; 5 CAS Key Laboratory of Genome Sciences and information, Beijing Institute of Genomics of CAS, Beijing, 100101 People’s Republic of China; 6 Department of Bioinformatics, School of Life Sciences and Technology, Tongji University, Shanghai, China

**Keywords:** *Cis*-regulatory motif, Phylogenetic footprinting, Prokaryotic genomes, Comparative genomics

## Abstract

**Background:**

Phylogenetic footprinting is an important computational technique for identifying *cis*-regulatory motifs in orthologous regulatory regions from multiple genomes, as motifs tend to evolve slower than their surrounding non-functional sequences. Its application, however, has several difficulties for optimizing the selection of orthologous data and reducing the false positives in motif prediction.

**Results:**

Here we present an integrative phylogenetic footprinting framework for accurate motif predictions in prokaryotic genomes (MP^3^). The framework includes a new orthologous data preparation procedure, an additional promoter scoring and pruning method and an integration of six existing motif finding algorithms as basic motif search engines. Specifically, we collected orthologous genes from available prokaryotic genomes and built the orthologous regulatory regions based on sequence similarity of promoter regions. This procedure made full use of the large-scale genomic data and taxonomy information and filtered out the promoters with limited contribution to produce a high quality orthologous promoter set. The promoter scoring and pruning is implemented through motif voting by a set of complementary predicting tools that mine as many motif candidates as possible and simultaneously eliminate the effect of random noise. We have applied the framework to *Escherichia coli* k12 genome and evaluated the prediction performance through comparison with seven existing programs. This evaluation was systematically carried out at the nucleotide and binding site level, and the results showed that MP^3^ consistently outperformed other popular motif finding tools. We have integrated MP^3^ into our motif identification and analysis server DMINDA, allowing users to efficiently identify and analyze motifs in 2,072 completely sequenced prokaryotic genomes.

**Conclusion:**

The performance evaluation indicated that MP^3^ is effective for predicting regulatory motifs in prokaryotic genomes. Its application may enhance progress in elucidating transcription regulation mechanism, thus provide benefit to the genomic research community and prokaryotic genome researchers in particular.

**Electronic supplementary material:**

The online version of this article (doi:10.1186/s12864-016-2982-x) contains supplementary material, which is available to authorized users.

## Background

Identification of regulatory DNA motifs represents a fundamental step in the study of transcriptional regulation mechanisms. Regulatory motifs typically facilitate the gene transcriptional regulation as transcription factors binding sites (TFBSs). Computational prediction of motifs in promoters has evolved as an increasingly important problem since it was proposed in 1980s [1–3]. In the past three decades, a number of programs have been developed such as AlignACE, Biprospector, CONSENSUS, MDscan, MEME, CUBIC and BOBRO [4–13]. In spite of the substantial number of applications that have been developed, it is still a very challenging problem and there is much room for improvement in motif identification performance [2, 3, 14, 15].

The phylogenetic footprinting strategy, first proposed by Tagle et al. in 1988 [16, 17], has proven useful in *de novo* motif finding. This strategy is based on a common principle that the regulatory elements in promoters tend to evolve at a lower rate and be more conserved at the DNA sequence level than their surrounding non-functional sequences. Following this line of research, scientists first applied comparative genomics methods [18] and co-regulation based motif finding tools on orthologous promoters to detect regulatory signals. Later, specific tools for phylogenetic footprinting [19–24] were designed to improve the performance of motif identification. In the last decade, with the increased availability of sequenced prokaryotic genomes and the sequence-similarity based orthology mapping technology, researchers have made application of phylogenetic footprinting less difficult and more powerful [25].

However, the application of phylogenetic footprinting is still intractable for researchers, because almost all existing methods require several tough procedures. Many factors need to be considered for proper phylogenetic footprinting application use, such as reference species selection, orthology mapping and promoter region cutting [15]. The noise induced by each of these factors can increase motif prediction false positives. Further the promoters generated for a set of orthologous genes should be divergent enough so that the to-be-identified motifs stand out, yet limit the mutations, thus maintaining the conserved motif properties. Specifically, phylogenetic footprinting applications have the following limitations [16]: (i) Lack of reliable genome-scale operon structure integration, which is essential for regulatory motif prediction in prokaryotes [26, 27]; (ii) Lack of universally applicable promoter collecting framework, which makes full use of abundant sequenced genome data. (iii) Neglecting to identify the phylogenetic relationship among promoters. (iv) The need for users to set poorly-defined motif feature parameters or other algorithmic thresholds. (v) Lack of intuitive and user-friendly tools or web server, although some methods have been proven effective on biological data sets. Most users do not understand how to adjust these factors and application parameters to ensure accurate motif prediction.

In this paper, we propose a framework for Motif Prediction based on Phylogenetic footprinting (MP^3^) (Additional file 1: Figure S1), aiming to avoid the drawbacks described above and make the pipeline effective and widely applicable. New strategies were developed for (i) integrating the sequence-similarity and functional association information in orthologous promoter selection, (ii) promoter scoring and pruning through motif voting using a set of complementary predicting tools and (iii) motif signal cross validation using a curve fitting method. We validated MP^3^ using the whole genome of *E. coli* K12, which has many documented TFBSs in RegulonDB [28]. The performance was systematically evaluated and compared with seven other existing tools. The comparisons show that MP^3^ has significantly improved performance over other existing tools. We implemented MP^3^ into a stand-alone program, which is available at http://csbl.bmb.uga.edu/DMINDA/download.php. Furthermore, the whole pipeline has also been implanted into DMINDA (http://csbl.bmb.uga.edu/DMINDA/) [29], which is an integrated web server for DNA motif prediction and analyses based on our in-house motif identification programs BOBRO [5, 30] and the DOOR2.0 database containing operons for 2,072 prokaryotic genomes [27]. DMINDA allows MP^3^ to be readily applied on any of the 2,072 integrated prokaryotic genomes and provides a user-friendly platform for visualization and display of the prediction results.

## Methods

**Fig. 1:**
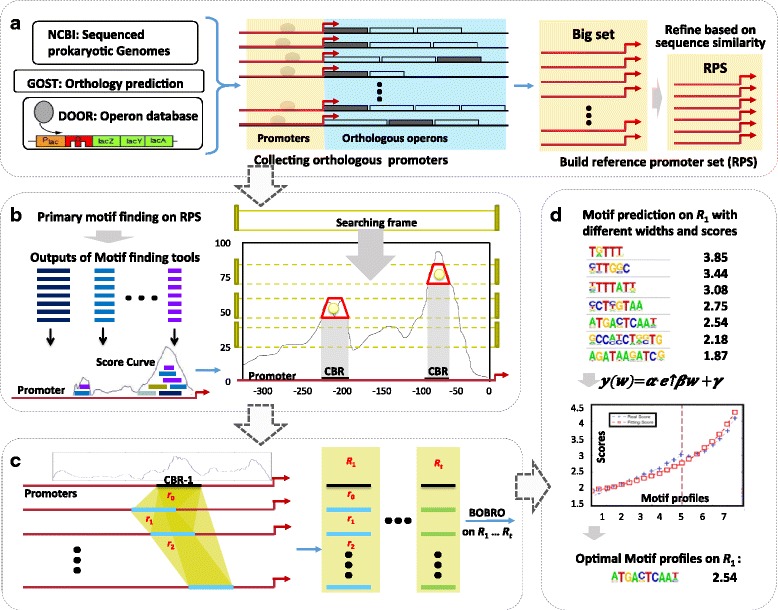
An outline of the MP^3^ framework. **a** RPS preparation based on sequenced genome from NCBI, operon information retrieved from DOOR, and identified orthologous genes for a target gene using GOST. The promoters of orthologous operons are generated and then are refined to build RPS. **b** CBR detection by voting strategy and peak finding. The predicted motifs by six tools (short sequences) are mapped back on promoter sequences, and generate score curves. The peaks on the curve are identified as CBR by a peak calling method. **c** CBR clustering based on a new graph model. *r*_0_, *r*_1_… are CBRs on promoters, which are clustered together as a related CBR set *R*_1_. The motif finding will performed on these clusters (*R*_1_, *R*_2_, …, *R*_*t*_) again to build motif profiles. **d** Motif profiles identification and motif width optimization through curve fitting

MP^3^ has four components: reference promoter set (RPS) preparation from sequenced prokaryotic genomes (Fig. 1a), candidate binding region (CBR) detection by motif voting strategy and peak finding (Fig. 1b), candidate binding region clustering based on a graph model (Fig. 1c), and motif profile identification through curve fitting (Fig. 1d).

### Preparation of reference promoter set (RPS) of a given gene in MP^3^

*Collection of orthologous promoters*: The traditional strategy for orthologous gene collection in phylogenetic footprinting relies on choosing several species in advance [15, 25, 31, 32]. This can limit the quantity and quality of available orthologous genes. MP^3^ collects the orthologous genes from a large set of references genomes, i.e. “*big data source*”. Specifically, (i) we used the recent orthology detection tool, GOST [33] to identify the orthologous genes of any given prokaryotic gene in the reference genomes. These genomes belong to the same phylum, but a different genus than that of the target gene, and we took only one genome into consideration for each genus to avoid redundancy. We (ii) then extended the orthologous relationship from gene to operon level. Thus, for a given gene, its host operon is denoted as *o*_0_ = {*g*_1_, *g*_2_,…, *g*_*r*_}(*r* ≥ 1) and the operons in the reference genomes that contain orthologous genes of any *g*_*i*_ in *o*_0_ (*i* = 1, …, *r*) are considered as orthologous operons of *o*_0_, denoted as {*o*_1_, *o*_2_, …, *o*_*n*_}. Their promoter sequences are defined as corresponding upstream regulatory regions (up to 300 bp), denoted as *p*_0_ and {*p*_1_, *p*_2_, …, *p*_*n*_}, respectively. Then iii), we define the promoter set *P =* {*p*_1_, *p*_2_, …, *p*_*n*_} as the orthologous promoters of *p*_0_.

*Reference Promoter Set (RPS)*: The preliminary orthologous promoter set obtained above could not be directly used to predict motifs, as the large data set size and unconsidered phylogenetic relationships can overpower the conserved motif signal. MP^3^ polished the preliminary promoter set to generate a reference promoter set (RPS), which was of reasonable size and with conserved significant motifs, i.e. “*reduced final set*”. Our selection strategy was partly inspired by McCue et al., who claimed that three well-selected reference promoters might be sufficient to identify a motif on a given human gene [15]. We improved this model for application in prokaryotes by selecting three groups of orthologous sequences instead of just three sequences. In addition, rather than using existing phylogenetic tree based on species, phylogenetic trees were assembled for each group of orthologous promoters. Before selection, the phylogenetic tree of orthologous promoter sequences was built by ClustalW [18], and the distance scores of this tree were used to represent the distance between any pair of orthologous promoter sequences. MP^3^ then divided *P* into three groups, *P*^1^, *P*^2^, and *P*^3^, corresponding to highly similar to, relatively similar to, and distant from *p*_0_, according to the thresholds obtained by analyzing the distribution of distance scores between orthologous promoters (Additional file 1: Method S1 and Figure S2). MP^3^ first selected three reference promoters from each group, and then added three more from *P*^3^, because *P*^3^ has many more orthologous promoters. In this selection, we considered the additional following factors: (i) The promoters whose operons had the same leading orthologous genes with *O*_0_ had higher priority to be chosen. (ii) The promoters were re-ranked based on a genomic similarity score (GSS) [33], which was calculated as the fraction of genes in the target genome, which have orthologous genes in the reference genome. We selected promoters with higher GSS based on the assumption that the genome with higher GSS tends to have regulatory mechanism more similar to that of the target genome [15]. (iii) Any two selected promoters were required to have a mutual distance score greater than 0.05 to avoid redundant promoters. Finally, the selected reference promoters, along with *p*_0_ itself, composed a reference promoter set (RPS), which was expected to contain key motif signals and have a reasonable size with the consideration of computational efficiency. More details about RPS generation are provided Additional file 1: Method S1.

### Pruning promoter to identify *Candidate Binding Region* (CBR)

For a given gene, the RPS can be used to prune its corresponding promoter *p*_0_ and identify rough TF binding regions through a voting strategy by integrating multiple motif finding tools (Fig. 1b). Six widely used *de novo* motif finding tools, Biprospector, BOBRO, MDscan, MEME, CUBIC, and CONSENSUS [4, 5, 8–11], were applied to the RPS to identify conserved motifs with lengths ranging from 5 to 30, and for each length, we kept the top ten predicted motifs (if available). The predictions for a specific program can be denoted as

1 $$ S={\displaystyle \underset{l=5}{\overset{30}{\cup }}}{\displaystyle \underset{t=1}{\overset{10}{\cup }}}\kern0.5em {S}_{lt} $$

where *S*_*lt*_ represents the *t*-th motif in the prediction with length *l*. If *S*_*lt*_ contains an instance from *p*_0_, denoted as *s*, its contribution will be added to the voting score *C*_*i*_ (set to 0 initially) using the following formula (Fig. 1b),

2 $$ {C}_i\kern0.5em =\kern0.5em {C}_i\kern0.5em +\kern0.5em {V}_s,\kern0.5em \mathrm{f}\mathrm{o}\mathrm{r}\kern0.5em i\kern0.5em \in \kern0.5em \left\{i\Big|{b}_s\le i\le {e}_s\right\}; $$

where *b*_*s*_ and *e*_*s*_ represent the starting and ending positions of *s* along *p*_0_, and

3 $$ {V}_s\kern0.5em =\kern0.5em \frac{1}{\left|{S}_{l\bullet}\right|\left(1\kern0.5em +\kern0.5em  \log t\right)},\kern0.5em {S}_{l\bullet}\kern0.5em =\kern0.5em \underset{t=1}{\overset{10}{\cup }}{S}_{lt} $$

where *t* is the rank of motif profile, which motif instance s belongs to, in prediction results for input length *l*. Intuitively, such voting scores are reliable and informative as different tools do have complementary effects [6, 14] while the false positive noise tend to randomly distribute in *p*_0_. The voting scores generally represent the support obtained from multiple predictions. The larger a score, the higher probability that the site overlaps true TFBSs. Additionally, we normalized the contribution of different predictions by introducing *S*_*l*_., instead of directly counting the number of predicted segment covering each site, since the output size of motif finding tools may be very different.

Application of a pick calling strategy to the voting scores allows a set of CBRs to be identified, each of which is recognized as a continuous genomic segment of *p*_0_, containing nucleotides with significant higher voting scores than the surrounding sequence. Additional details can be found in Additional file 1: Method S2. The CBRs, as primary output of MP^3^, can be used by researchers directly in genetic engineering to locate the functional regulatory regions of a promoter.

### Clustering of correlated CBR set

The CBR sets identified in the target and reference promoters are used to build motif profiles (Fig. 1c). A similarity graph *G* with all CBRs represented as vertices and edges connecting every pair of vertices was constructed. The weight of edges are set as the correlation scores between two corresponding CBRs as follows: (i) *p*_0_ and *p*_1_ are the target promoter and a reference promoter, respectively; (ii) a CBR *c*_0_ in *p*_0_ begins at *b*_0_ and ends at *e*_0_ (−|*p*_0_| ≤ *b*_0_ < *e*_0_ ≤ −1) and another CBR *c*_1_ begins at *b*_1_ and ends at *e*_1_ in *p*_1_ (the start of coding regions as the origin position 0). (iii) the correlation score *W*(*c*_0_, *c*_*j*_) between the two CBRs was evaluated:

4 $$ W\left({c}_0,{c}_1\right)=\left(1-\frac{\left|{b}_0-{b}_1\right|}{ \max \left\{\left|{b}_0\right|,\left|{b}_1\right|\right\}}\right)\times S\left({c}_0,{c}_1\right) $$

where *S*(*c*_0_, *c*_1_) was the sequence similarity score, calculated by aligning *c*_0_ and *c*_1_. The weight of the edge that connects CBRs of the same promoter will be set as 0. Clearly, the higher a weight, the more correlated the two corresponding CBRs were. The relative location of CBR pairs *S*(*c*_0_, *c*_1_) was also considered as the position of many TFBSs tend to be conserved in evolution [34].

Intuitively, a set of highly correlated CBRs should be connected by large weights producing a subgraph of *G*, i.e. subgraph with large edge weight, because these correlations should make the weight of each involved edge larger. It should also be noted that identifying all heavy subgraphs in a weighted graph itself was NP-hard. Hence, we identified the CBR clusters in a heuristic way: (i) we sorted the edges in *G* in decreasing order of their weights and only keep the top 1/3. One third was absolutely enough because the graph with only real connections should be sparse. However, the random cliques have little chance to survive because graph *G* is a multi-partite graph; (ii) we obtained the induced sub-graph of a CBR in target promoter and its neighbors in other promoters; and (iii) we detected the maximal clique in induced sub-graph and then expanded it by including the highly connected vertex. The CBRs corresponding to the vertex in each cluster composed the correlated CBR set in which the motif profile identification will be carried out.

### Identification of candidate motif profiles

*Building Motif profiles from correlated CBR set*. We applied our motif finding tool, BOBRO [5] on the identified CBR sets to generate candidate motif profiles. Outstanding motif instances were identified using the support from several motif finding tools (Fig. 1d).

It was still very challenging to evaluate motif profiles with different widths. Although BOBRO and MEME are capable of detecting motif width on co-regulated promoters, they may fail on phylogenetic footprinting data, because the flanking regions of motifs in orthologous promoters are usually conserved to some extent. In MP^3^, a curve fitting method was designed to detect the motif profiles with an optimized width for phylogenetic footprinting. The BOBRO predicted motif profiles have a width from 6 to 22 and corresponding IC (information content) scores, which are calculated by the formula:

5 $$ IC(w)={\displaystyle \sum_{j=1}^w}{\displaystyle \sum_{i=1}^4}{f}_{ij}lo\mathit{\mathsf{g}}\frac{f_{ij}}{b_i} $$

where (*f*_*ij*_) is the probability of nucleotide type *i* appearing at position *j* in the motif profile, and *b*_*i*_ is the probability of *i* appearing in the background sequence which is calculated on all input promoter sequences. However, IC cannot be directly used to compare different motif profiles, because they are width-dependent. MP^3^ regresses the correlation function between the IC and the width of motif profile by minimizing

6 $$ \underset{w=\kern0.5em 6}{\overset{22}{\varSigma }}{\left[IC(w)\kern0.5em -\kern0.5em f(w)\right]}^2 $$

on the conjectured function:

7 $$ f(w)\kern0.5em =\kern0.5em a\kern0.5em \cdot \kern0.5em {e}^{\beta w}\kern0.5em +\kern0.5em \gamma $$

where *α*, *β* and *γ* are fitting coefficients. Then, we took the difference between the real IC scores and fitting scores for each profile, i.e. the residual of above regression,

8 $$ r(w)\kern0.5em =\kern0.5em IC(w)\kern0.5em \mathit{\hbox{-}}\kern0.5em f(w) $$

as the criterion to select the best motif profile. Basically, the motif profiles whose *r*(*w*) are local maximum are ranked in the decreasing order of *r*(*w*).

### MP^3^ application and performance evaluation using *E. coli* genome

**Fig. 2:**
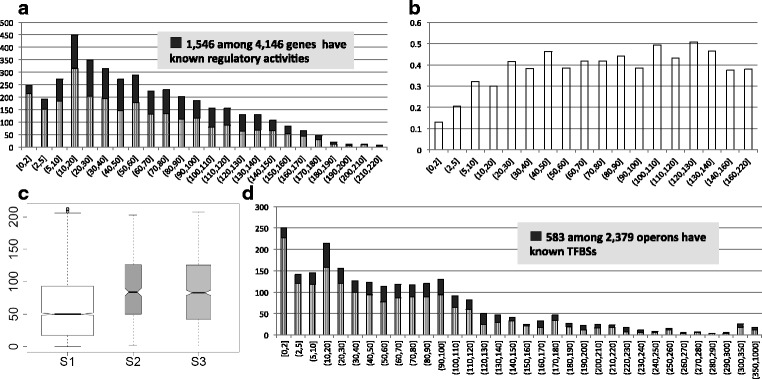
The information about genes, orthologous, regulatory activities, and promoters. **a** The distribution of orthologous gene number: The *x*-axis is the number interval of orthologous genes; the *y*-axis is the number of genes whose orthologous number is in the corresponding interval. The solid parts represent the genes having known regulatory activities. **b** The correlation between orthologous number and regulatory activities: The *x*-axis is the number interval of orthologous genes; the *y*-axis is the proportion of genes with known regulatory activities in corresponding gene groups. **c** The box-plot of orthologous number distribution for gene sets S1, S2 and S3. S1 represents the whole gene set of *E. coli*; S2 and S3 are the central metabolism genes and all pathway genes respectively. The genes in S2 and S3 have significantly more orthologous compared to S1 with Wilcox p-values both as 2.2e-16, and the genes in S2 have little more orthologous than S3 with Wilcox p-value as 0.17. **d** The distribution of orthologous operon number: The *x*-axis is number interval of orthologous operons; and the *y*-axis is the number of operons whose orthologous number within corresponding intervals. The solid parts represent the operons having known TFBSs in regulatory regions

*Data Acquisition*. We used *E. coli* K12 as the target genome and another 216 selected prokaryotic genomes from the Proteo-bacteria phylum as references to test MP^3^ methods and the applications. The genome data were downloaded from the NCBI database (released as of November 2011). The 216 reference genomes were obtained from 216 different genera (a general principal for orthologous data for MP^3^) to avoid potential selection bias in comparative genomics studies [33]. The operons of these genomes were retrieved from the DOOR2.0 operon database [27, 35], and the documented motifs in *E. coli* were obtained from RegulonDB [28]. We linked the documented TFBSs in *E. coli* to their target operons and then to corresponding promoters in the identified 2,252 RPSs. Figure 2d showed that 583 of the 2,379 operons have experimentally confirmed TFBSs (solid bars in black) in their regulatory regions. Twenty of these 583 operons and their corresponding TFBSs were removed since they did not have enough orthology. The remaining 563 promoter sequences, containing 2,048 binding sites, were used to evaluate the performance of MP^3^. Besides, we downloaded Sigma 70 binding promoters of *E. coli* from the RegulonDB and conducted analysis to see the correlation between orthology and Sigma 70 binding in *E. coli*.

*Performance evaluation*. To conduct performance comparison, we applied six *de novo* motif finding tools previously mentioned, i.e., Biprospector, CONSENSUS, MDscan, MEME, CUBIC, BOBRO and a phylogenetic footprinting pipeline MicroFootprinter [4–13, 21, 25, 30, 36] on the same genome and compared with MP^3^. We followed Tompa’s method [14] and assessed the predictions both at nucleotide level and at the binding site level. Specifically, we calculated the sensitivity (nSN), positive prediction value (nPPV), specificity (nSP), performance coefficient (nPC) and correlation coefficient (nCC) at nucleotide level, and calculated the sensitivity (sSN), positive prediction value (sPPV), and average site performance (sASP) at site level. In addition, we added the widely used F-score (sFS) at site level for better evaluation. The calculation details for these measures can be seen in Additional file 1: Method S3. We followed Tompa’s criterion to indicate that a predicted site overlaps a known TFBS if they overlapped by at least 1/4 the length of known site [14].

### Functional enrichment analysis according to the KEGG database

For a set of operons in *E. coli*, we did functional enrichment analysis of the corresponding genes with DAVID [37]. Specifically, given a set of operons, their genes were picked from the DOOR2 database [27] and submitted to DAVID as the input gene list with this genome as background genome. The *p*-values were calculated in terms of a Bonferroni-corrected modified Fisher's exact test under the null hypothesis that this set of genes was not enriched with certain biological functions.

## Results

MP^3^ was applied on all the 4,146 genes of *E. coli* K12, with all the documented TFBSs from the RegulonDB database. The unique features of MP^3^ resulted in a positive effect in motif finding: the new strategy for orthologous promoter sequences selection makes phylogenetic footprinting efficiently applicable on most of prokaryotic genes, e.g. 90.5 % (2,252 out of 2,379) of *E. coli* operons have at least three orthologous operons. The promoter pruning method with motif voting and peak calling reduced the false positive rate, the positive prediction value increased from 0.43 to 0.584 and the F-score increased from 0.191 to 0.306 in performance evaluation on binding site level. The curve fitting for motif width optimization in the last step helped to build high quality motif profiles. In addition, with implementation of MP^3^ in DMINDA, users can obtain the motif prediction by simply clicking the name of a gene from each of the 2,072 prokaryotic genome in our back-end database and conduct further analyses (e.g. motif comparison, motif clustering, and motif co-occurrence analysis) for predicted motifs on the DMINDA platform.

### Orthologous repertoires of genes in *E. coli* K12 and their properties

For all 4,146 *E. coli* genes, 250,804 orthologous gene pairs between *E. coli* and each of the 216 reference genomes were identified by GOST. The distribution of the number of orthologs for all the target genes, ranging from 0 to 216, represents a huge difference from gene to gene (Fig. 2a). It indicated that the widely used species selection method, i.e. choose a few species before ortholog generation, may fail to obtain enough orthologs. Furthermore, this observation raised two questions: Is there any correlation between ortholog number and its transcriptional regulation mechanism for a specific gene; and what kinds of genes have more orthologs than the others? The answers to these questions may guide the application by identifying which genes are more suitable for the phylogenetic footprinting strategy.

*Gene’s transcriptional regulation is correlated with the number of its orthologous genes*. The RegulonDB database showed that 1,546 genes are regulated by one or more TFs, among all the 4,146 genes defined as known *regulatory activities* in our study. All 4,146 genes were divided into 18 groups according to the number of orthologous genes they contain (Fig. 2b). The results indicated that the genes with moderate number of orthologs tended to have more confirmed regulatory activities, while the genes with many or few orthologs tended to have less known regulatory activities. We hypothesize that the genes with more orthologs play essential function in cell, thus tend to keep a consistently high expression level and probably need less regulation. We also analyzed the correlation between Sigma70 binding motifs and the number of orthologs on operon level, and found that the operons with more orthologs tend to have Sigma 70 binding motifs (Additional file 1: Result S1 and Figure S3). This finding confirmed our hypothesize as Sigma 70 factors keep essential genes and pathways operating as a “housekeeping” sigma factor [38]. Meanwhile, genes with few orthologs usually have a specific function in their host genome; therefore, have both simple and specific regulation. In contrast, genes with a moderate number of orthologs have more responsibilities in biological diversity and have more regulation activities.

*Genes having more orthology information tend to be functionally necessary*. We ranked all operons in the decreasing order by their number of orthology and took the top 100 for functional annotation analysis according to the KEGG database [39]. The results showed that the most enriched function among them is Ribosome, which is the most important and essential function in any organism (Additional file 1: Table S1). The analysis also showed that the genes involved in known metabolic pathways (especially those in central metabolism) according to KEGG database do have significantly more orthologs compared to the others (Fig. 2c).

### Generation of 2,252 RPSs for *E. coli* K12 operons

The 4,146 genes in *E. coli* genome fell into 2,379 operons according to the DOOR2.0 database, giving rise to 2,379 target promoters (Table 1). The 250,804 orthologous gene pairs, between *E. coli* and reference genomes, were extended to 195,518 orthologous operon pairs, to facilitate the orthologous promoter sequences extraction. 90.5 % (2,252 out of 2,379) of *E. coli* operons have at least three orthologous operons with the average number as 81.1 (Fig. 2d), indicating that phylogenetic footprinting can be applied on most of prokaryotic genes. The rapid growth of genomic sequences from multiple organisms will further enhance the reliability of this large-scale search strategy. For 332 out of 2,252 operons (14.7 %), we simply added all orthologous promoters to their RPSs, as they had no more than 12 orthologous operons. Regarding the other 1,920 operons (85.3 %), MP^3^ builds the RPSs with the goal to compress promoter set without losing significance of conserved motifs (see details in Methods). Finally, we obtained 2,252 RPSs, containing an average of 11.3 reference promoters.

**Table 1:** The summaries of orthologous and motif prediction on *E. coli* K12 by MP^3^

Statistics on orthologous and prediction					
Genes				4,146	
Genes with known regulatory activities				1,546	
Average number of orthologous genes				60.49	
Operons				2,379	
Operons with more than 2 orthologous operons				2,252 (90.5 %)	
Average number of orthologous operons				81.1	
Promoter sequences				2,252	
Operons with known TFBSs				583	
CBRs by MP^3^				12,820	
Motif profiles by MP^3^ (Alternatives)				12,820 (76,732)	
Data in evaluation					
Promoter sequences with known TFBSs				563	
The known TFBSs				2,048	
Evaluation results on 563 promoters					
CBRs by MP^3^				3,205	
Motif profiles by MP^3^ (Alternatives)				3,205 (22,388)	
Top CBRs	1	2	3	4	5
CBR coverage	455 (22 %)	710 (35 %)	925 (45 %)	1,080 (53 %)	1,206 (59 %)
Motif Profiles coverage	425 (21 %)	675 (33 %)	878 (43 %)	1,022 (50 %)	1,133 (55 %)

### Prediction of conserved motifs in *E. coli* K12

In total, MP^3^ generated 12,820 CBRs for the 2,252 promoters, i.e., averagely 5.7 CBRs per target promoter (Table 1). A total of 93 % of the CBRs have length from 14 to 22 bps, which are associated with the width of peaks on the voting curve; while some CBRs are longer than average, which may be caused by the overlap of multiple binding sites in the promoters. For those 563 promoters with known TFBSs, 3,205 CBRs were identified. If we only considered the top CBR for each promoter, the 563 CBRs cover 455 known TFBSs, i.e., an average of three TFBSs for four promoters, thus a high accuracy with low false positives. However, the 455 TFBSs only accounted for 22 % of all 2,048 binding sites. This was mainly because many operons are regulated by multiple TFs and have multiple TFBSs. So it was worthwhile to consider more CBRs to better elucidate the motif information. We found that the top 5 CBRs cover 1,133 known TFBSs (55 % of all) and simultaneously brought more false positives. MP^3^ built motif profiles from all the 12,820 CBRs and output those with the highest confidence level from each by a curve fitting method, i.e. 12,820 motif profiles. These profiles can be used to identify new binding sites in other promoters or detect co-regulated operons through motif comparition.

### Performance comparison with existing motif-finding tools

We compared the prediction of MP^3^ with six *de novo* motif finding tools: BOBRO, MDscan, Bioprospector, MEME, CONSENSUS, CUBIC, and MicroFootprinter. MicroFootprinter is designed for phylogenetic footprinting on prokaryotic genomes and can generate orthologous promoters on its web-server; MDscan is designed for motif-finding on ChIP-Chip data; and the others are general *de novo* motif-finding tools. We chose default parameters for each of them, because the comparison was performed on the genome scale thus it was unrealistic to specifically adjust parameters for each individual gene in a trial-and-error way. The prediction results of MicroFootprinter were obtained from its web server manually, and it gave valid prediction only for 114 promoters among all 563 promoters with known TFBSs. The other six tools were tested on the RPSs identified by our framework, since applying *de novo* motif finding tools directly on a rough promoter sequence set is obviously naïve and unreliable.

**Fig. 3:**
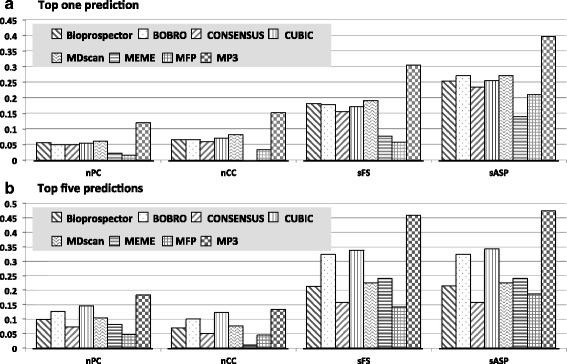
Representative statistics comparing the accuracy of MP^3^ with other tools. The statistics in (**a**) and (**b**) are calculated by taking top one and top five prediction into consideration correspondingly

Using MP3 and seven other tools, we calculated nPC, nCC, sFS and sASP according to their best output (Fig. 3a). Unlike sensitivity or specificity, these measures were capable of evaluating the overall performance of prediction. The comparison showed that MP^3^ outperformed by 98 % in nPC, 88 % in nCC, 60 % in sFS and 46 % in sASP over MDscan, which is the best of the other seven tools. There are on average 2.8 TFBSs for each of 563 promoters according to known TFBS, and only a fraction of TFBSs have been documented. Therefore, we further compared the performance of these tools on their top five predictions. In this case, the improvement made by MP^3^ over the best one of other seven tools (CUBIC) are 25.3 % in nPC, 8.1 % in nCC, 35.7 % in sFS and 38.6 % in sASP. It is worth noting that, even though MicroFootprinter provides much fewer results, its predictions have higher specificity. MDscan had a relatively higher performance than the other published tools. MDscan starts on an enumeration strategy on the top several sequences, which is more adaptable to the data of phylogenetic footprinting motif finding. Additional performance statistics can be seen in Additional file 1: Table S2.

### Performance bias of TFBSs prediction according to their different locations within a promoter

**Fig. 4:**
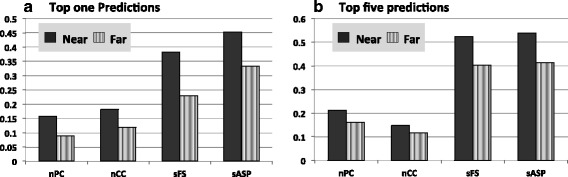
Performance comparison of MP^3^ on the near and far upstream region of target genes on the top one predictions (**a**) and top five predictions (**b**) correspondingly for each promoter

Interestingly, we found that MP^3^ has better performance for the documented TFBSs near their downstream genes than those far from their downstream genes. Specifically, we considered the −100 site upstream from the translation start site of a gene as a boundary, by which the whole intergenic region was divided into two parts. The region [−100, −1] is denoted as the *near* regions, and the other part of the intergenic region is called the *far* region. Then we did the similar performance evaluation as described in above Methods and Results section. The evaluation results showed that the performance was much better in detecting the binding sites in the *near* regions than in the *far* regions (Fig. 4 and Additional file 1: Table S3). We believe that the possible reasons for this bias could be: (i) the binding sites located in the *far* regions have greater probability to be regulatory elements of other neighboring genes, but were computationally assigned to the target gene in mistake; (ii) the specific binding mechanism of some TFs do not require constant binding location. Hence the distance between their binding sites and the target genes may be more flexible, thus easy to be missed by MP^3^, whose CMP clustering algorithm prefers the binding sites with constant locations.

It should also be noted that there are alternative transcription units inside the operons in prokaryote, and the motifs may be located on inner-operon no-coding regions [27, 28]. Hence, another issue in phylogenetic footprinting is how to deal with these non-coding regions within operons. Considering that these motifs account for only a limited fraction of the motifs, we simply ignored these regions in MP^3^ by default to reduce the potential noise induced by adding them. For the users who are interested in this kind of motif, we suggest they manually connect the inner-operon non-coding sequences on the tail of target promoter and carry out the same motif finding analysis on MP^3^ web-server to retrieve all the conserved motifs.

### MP^3^ Implementation in DMINDA

The whole pipeline of MP^3^ has also been implanted into DMINDA [29], which is an integrated web server for DNA motif prediction and analyses using our in-house motif identification program BOBRO [5] and the DOOR2.0 database containing operons for 2,072 prokaryotic genomes. We listed all genes for the 2,072 prokaryotic genomes and the orthologous promoter were collected using the same method on *E. coli*, thus users can perform this proposed motif finding framework on them in several clicks. Current motif-related tools implanted in DMINDA, e.g. motif scanning and comparing, are available to assist the users needing to use other protocols beyond the motif prediction for specific biological hypotheses. Details about the implementation of MP^3^ in DMINDA can be seen in Additional file 1: Result S2 & Figure S4.

## Discussion

The phylogenetic footprinting technique has several intrinsic limitations in *de novo* motif finding. For example, it cannot be used on genes that have almost no orthology in other sequenced genomes; and it is incapable of identifying TFBSs that have no conservation properties at the sequence level (i.e., lack of sequence specificity) [40]. Lateral gene transfer and operon structure exist widely throughout prokaryotic genomes unlike in vertebrates. Therefore, direct use of the species tree and the phylogenetic tree inferred from the targets genes, as done in current published methods, is not the best choice for prokaryotic genomes [25]. However, an improved phylogenetic footprinting method would be useful as it also has important applications for elucidating the underlying gene regulatory networks [41]. Recently, Novichkov et al. proposed an algorithm Regpredict to generate regulons, which are defined as maximal co-regulated gene sets [42, 43]. Regpredict takes advantage of phylogenetic footprinting to reduce the false positives, thus improves the reliability of predicted regulon on multiple genomes.

MP^3^ was developed to overcome the drawbacks of the existing phylogenetic footprinting tools. The MP^3^ framework (Fig. 1) has the following unique features: (i) full consideration of the operon structures; (ii) new promoter collection method following a principle named as *big data source, reduced final set*, which not only takes advantage of high throughput genomic data, but also considers the computational efficiency; (iii) extracting phylogenetic relationship from regulatory sequences to refine the orthologous promoter set. (iv) pruning promoters to generate CBRs based on the weighting score on each nucleotide, which is generated by a voting strategy on six popular motif finding tools; and (v) a curve-fitting method to identify optimal motif profiles. Based on these features, MP^3^ had a much better performance in motif finding.

For our new phylogenetic footprinting pipeline, a potential and reasonable improvement is integrating some experimental data, if available, e.g. Chromatin immunoprecipitation followed by sequencing (ChIP-seq). It is a technique used for genome-wide profiling of DNA-binding proteins, histone modifications, or nucleosomes; and has become an indispensable tool for studying gene regulation [44, 45] as it can provide transcription factor binding information with higher resolution, less noise, and greater coverage than traditional array-based predecessor, like ChIP-chip [46]. However, it cannot replace the computational prediction tools particularly for prokaryote. Firstly, there is very small amounts ChIP-seq data available for prokaryote [47]; secondly, ChIP-seq is not suitable for TFs with only a few binding sites; thirdly, the complexity of regulation can also lead to bias because TFs may not bind on their binding sites in certain environments. Specifically, the score curves used in MP^3^ can be further optimized by integrating the binding signal from ChIP-seq, using machine learning or pattern classification. The ChIP-seq based peaks and CBRs identified by MP^3^ can be cross-validated by each other in application, aiming to overcome some intrinsic computational challenges in high-throughput data analyses. Upon the availability of large-scale ChIP-seq data in prokaryote [47], we believe that the information integration in our framework can further improve the performance in motif prediction and analysis.

An intuitive application of the MP^3^ motif prediction pipeline is to elucidate the genome-scale transcription regulatory network, which is one of the most important goals in systems biology. It can help infer how gene regulatory networks will respond under various conditions or with specific genetic perturbations; and to understand how different gene expression states are controlled by their underlying regulatory systems. Mathematically, this is modeled as a *regulon* identification problem, aiming to identify all the co-regulated genes by each of regulatory transcription factors. We note that there is a limitation in the MP^3^ application. For predicted motif profiles, we found that the motif profiles composed by orthologous binding sites may not perfectly coincide with those composed by binding sites of co-regulated genes in the same genome. For example, the transcription factor ArgR has 25 known binding sites in *E. coli*. The orthologous binding sites from the promoters of gene argR and its orthologous showed high similarity with only eight out of the 25, thus the motif logos have some differences (Additional file 1: Figure S5). The reason for this phenomenon may lie in the evolution mechanism for binding sites. The differences in orthologous binding sites are caused by heredity while the binding sites upstream of co-regulatory genes may be caused by gene duplication or even random mutation, thus leading to variation in these two motif profiles. The phenomenon described above may challenge the computational application and require additional algorithm development in motif based regulon construction.

## Conclusion

In this paper, we designed a new framework, MP^3^, for phylogenetic footprinting motif identification and provide it as a web service. The framework is based on several new ideas, integrated several existing motif finding tools, conquered the existing obstacles for orthology generation, false positive elimination etc. MP^3^ first generates CBRs, which may be directly used by researchers who only care to identify the functional regulatory regions of target genes; and then produces motif profiles for those that need motif profiles for motif search and comparison. The automatic pipeline of data acquisition, processing and implantation as web server allow easy application of MP^3^ to most sequenced prokaryotic genomes. Application on *E. coli* K12 genome in this study showed that MP^3^ worked better than existing motif finding tools and provides accurate results with less redundancy. We believe that MP^3^ will enhance progress toward elucidating the transcription regulation mechanism, especially for the genomes that have not been well studied. Thus, MP^3^ will benefit the genomic research community, and prokaryotic genome researchers in particular. In addition, using MP^3^ with other experimental techniques and knowledge will provide more reliable and useful results for regulatory research.

## Abbreviations

CBR, candidate binding region; ChIP-seq, chromatin immunoprecipitation followed by sequencing; IC, information content; MP^3^, motif prediction based on phylogenetic footprinting; nCC, correlated co efficient on nucleotide level; nPC, performance coefficient on nucleotide level; nPPV, positive prediction value on nucleotide level; nSN, sensitivity on nucleotide level; nSP, specificity on nucleotide level; RPS, reference promoter set; sASP, average site performance on site level; sPPV, positive prediction value on site level; sSN, sensitivity on site level

## Additional file

Additional file 1: Method S1-S3, Result S1-2, **Figure S1-S5**, **Table S1-S3**. (PDF 2276 kb)
