# Supplementary material for: An integrative and applicable phylogenetic footprinting framework for cis-regulatory motifs identification in prokaryotic genomes
Source: BMC Genomics. 2016 Aug 9;17:578. doi: 10.1186/s12864-016-2982-x (PMC4977642; doi:10.1186/s12864-016-2982-x)
Supplement: Additional file 1: — Method S1-S3, Result S1-2, Figure S1-S5, Table S1-S3. (PDF 2276 kb) [file 12864_2016_2982_MOESM1_ESM.pdf]

Supplementary Materials

An integrative and applicable phylogenetic footprinting framework for *cis*-regulatory motifs identification in prokaryotic genomes

Contents

**Fig. S1**	**2**
**Method S1**	**3-5**
**Fig. S2**	**5**
**Method S2**	**6**
**Method S3**	**7**
**Result S1**	**8**
**Fig. S3**	**8**
**Table S2**	**9**
**Result S2**	**10-11**
**Fig. S4**	**11**
**Table S3**	**12**
**Fig. S5**	**13**
**Additional References**	**14**
**Table S1**	**15-35**

Fig. S1

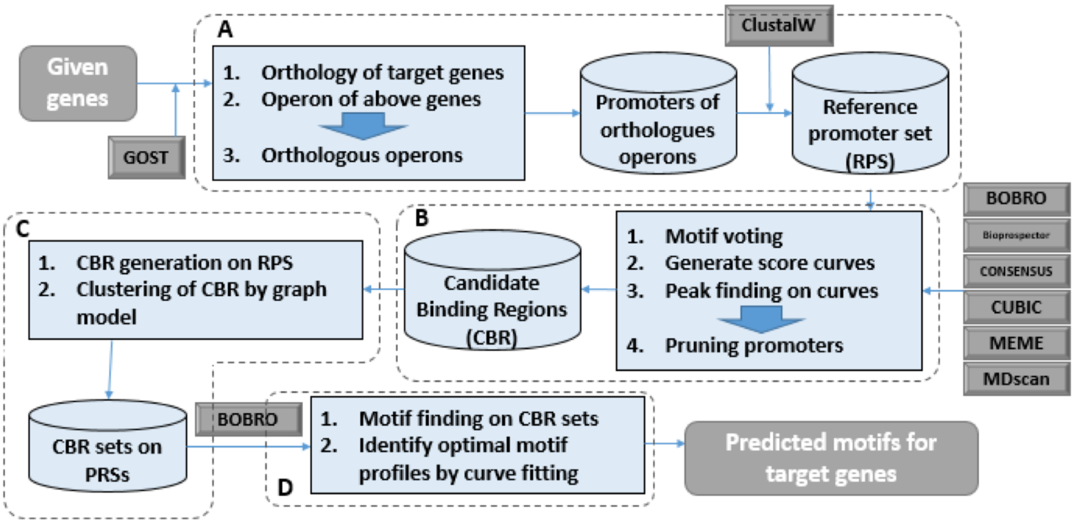

Fig. S1: The outline of MP^3 framework;

Method S1: generation of RPS from rough orthologous promoters

The collection of orthologous promoters is an essential step in phylogenetic footprinting. As discussed in main text, traditional strategy in orthologous genes collection for phylogenetic footprinting is choosing several species in advance [1-4], this usually limits both the quantity and quality of available orthologous genes, especially when applied to prokaryotes. The published methods usually apply motif finding tool directly on these rough orthologous promoters set. This is unreliable method of detecting motifs because both the improper data size and unconsidered phylogenetic relationships can drown the conserved motif signal. Improvements have been made by integrating phylogenetic tree, usually generated by comparison of 16s RNA or target orthologous genes. McCue. *et al* [3] said three well selected species may be sufficient for a given gene, that is, in proper distance from target gene. They indicated that three well-selected orthologous sequences could make the conserved motifs stand out and effectively detected by existing motif finding methods. These strategies worked well in Eukaryotes but may have problems in prokaryotes because of the widely existing horizontal gene transfer and operon structure in prokaryotic genomes.

Considering the intrinsic differences between prokaryotic and eukaryotic genomes, we improved the model by selecting three groups of orthologous sequences, corresponding to "close", "middle", and "far" comparing with target promoters, instead of three sequences. MP^3 uses an adapted strategy named "*huge data source and small final set*" to search as much gene orthology as possible. The abundant prokaryotic genomes, especially our in-house DOOR2 operon database, provide good opportunity to carrying out this strategy. This method allows the collection of better quality and quantity of orthologous gene sets. Then MP^3 filters the sets into a proper size with several properties (RPS), which benefit the following motif finding step. Two main principles were utilized in MP^3: (i) each individual promoter is valuable, and (ii) the composition is capable of making real binding sites significant enough. For (i), the search of orthology in abundant prokaryotic genomes guarantees that the valuable reference promoters will not be missed, and using sequence-similarity based method excludes the bad sequences.

Specifically, we use distance scores of promoter sequences on their phylogenetic tree, which calculated by ClustalW, to group orthologous promoters for each target into three subgroups (P^1, P^2, P^3). The reasons are that: 1) The phylogenetic tree on orthologous promoter sequences is more reliable for representing the evolution distances of the promoter region for a single gene than phylogenetic relationship generated by comparison of 16s RNA. 2) The new strategy can exclude the fake promoters caused by wrong operon information. The three thresholds (0.31, 0.55, and 0.72) are obtained by analyzing the distribution of distance scores between orthologous promoters (fig. S2). In figures, we show the distribution functions of similarity scores in three groups. Scores of group A are distances between the

promoters of target genes in *E. coli* and the promoters of their orthology; Scores in group B are pairwise scores in same orthology groups; and scores in group C are random background. Based on analysis on this figure, we found that the sequences with scores less than 0.55 hardly have chance to be random noises. Therefore, we take the first half (≤ 0.31) as "close orthologous promoters, i.e. P^1" and the second half (≤ 0.55 and >0.31) as "middle orthologous promoters, i.e. P^2". With the increasing of distance scores, the introduced sequences have little chance to be random ones, until the scores greater than 0.72. So, we take these promoters as "far orthologous promoters", and consider promoters with similarity score larger than 0.72 with target promoters as invaluable. Besides, promoters that are too similar with target promoters (with scores less than another threshold 0.05) will be considered as redundancy. The proportions of sequences in three groups were trained though experiments on several proportion schemes (Fig. S2B). The results proved that it would be better if we guaranteed every group was non-empty. We further found that the scheme 3-6-3 and 3-3-6 worked better than other schemes. Considering that the group P^3 had many more available sequences, we finally picked the scheme 3-3-6 in MP^3. In addition, in selection of the reference promoters, the promoters in each group were ranked based on a genomic similarity score (GSS) and the promoters whose operons have the same leading genes with target operon will be moved forward with the higher priority to be chosen.

For target promoter p_0 with its orthologous promoters $P=\{p_1, p_2, \ldots, p_n\}$, which is divided into three groups, P^1, P^2, and P^3. MP^3 built RPS for it in the following five steps:

Step 1. Put p_0 into RPS;

Step 2. Build the phylogenetic tree using p_0 and the sequences in P by ClustalW [5] and select reference promoters making use of their distance scores to p_0. In details, P was divided into three groups, P^1, P^2, and P^3, corresponding to highly similar to, relatively similar to, and distant from p_0, according to three intervals ([0.05-0.31], (0.31-0.55], and (0.55-0.71]) of the pair-wise distance scores with p_0 on phylogenetic tree;

Step 3. In each of the three groups, the promoters were re-ranked based on a genomic similarity score (GSS) [6] between their host genomes and the target genome in the increasing order;

Step 4. The promoters whose operons have same leading genes with O_0 have higher priority to be chosen;

Step 5. The top three, three, and six promoters (if any) from P^1, P^2, and P^3, respectively, were added to the RPS.

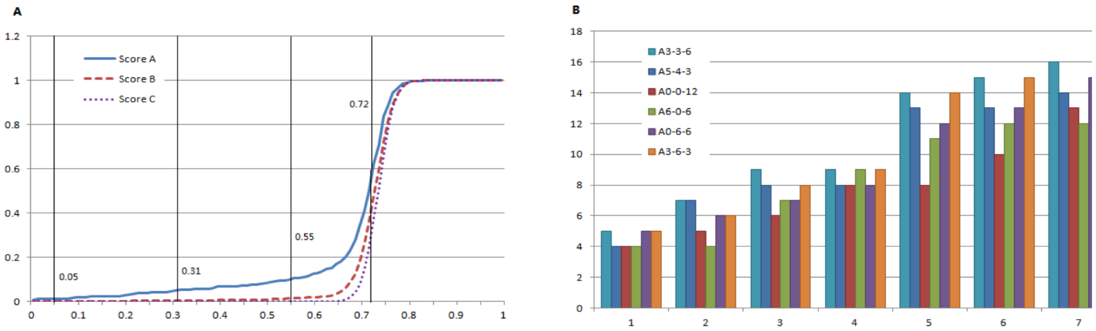

Fig. S2. The distribution of promoter similarity scores (A) and the performance of various sequence proportions (B). In A, the *x* axis is the similarity score, and the *y* axis is proportion of scores smaller than corresponding scores. The vertex lines on chart correspond to the thresholds for sequences filtering and groups assignment. In B, the *x* axis is different cut-offs for results involved in evaluation; the *y* axis is coverage rates for 6 proportion schemes. The label A3-3-6 means the final set has 3, 3, and 6 sequences from the 3 groups (P1 close, P2 middle, and P3 distant from target gene) respectively.

Method S2

The voting scores C_i can be seen as a curve along p_0, which will be used to identify CBRs on the target promoter sequences after being normalized to uniform scale. Basically, the CBR corresponds to the most significant peaks on the curve and we implanted a method in MP^3 to collect these peaks. Here, one peak is qualified if it is generally *high*, *steep*, and *wide* enough. Particularly, *high* means higher voting scores on the curve than its surrounding regions; *steep* means higher slope the peak has, which is controlled by two threshold ξ_1 and ξ_2 (0.5 and 0.25 in default) on the average of right slope and left slope; and *wide* means the peak fit the length of real motifs, usually ranging from 6 to 22 in prokaryote genome. Specifically, a two-layers searching frame with height d=5 and length covering whole promoter region will slide from top to bottom on the curve to detect peaks (see right diagram of Figure 1B). It worth noting that, the threshold ξ_1 and ξ_2 for slope evaluation and the height d of searching frame are heuristically selected based on the observation on real curves. Once a peak appears in frame, it will be dynamically evaluated based on the width and the average of right slope and left slope. In this up-to-bottom searching process, (1) Once the in-frame part of a peak has average slope greater than ξ_1, it will be labeled as primary candidate peak; (2) For a primary candidate peak, once its in-frame part has slope decreased to less than ξ_2, or has length longer than 22, which means that the peak is extending to flat regions or has been long enough respectively, it will be output as a picked peak. In addition, if two primary candidate peaks merge together during the frame going down, the new peak can be considered as primary candidate peak if any of them is a primary candidate peaks.

Method S3. The measures used in comparison and their values calculated on predictions by MP^3 and other seven tools.

For each tools, we calculate the statistics as Tompa did in his excellent assessment work[7].

- nTP is the number of nucleotide positions in both known sites and predicted sites;
- nFN is the number of nucleotide positions in known sites but not in predicted sites;
- nFP is the number of nucleotide positions in predicted sites but not in known sites;
- nTN is the number of nucleotide positions in neither known sites nor predicted sites;
- sTP is the number of known sites overlapped by predicted sites;
- sFN is the number of known sites not overlapped by predicted sites;
- sFP is the number of predicted sites not overlapped by known sites;
- Sensitivity on nucleotide level: nSN = nTP/(nTP+nFN);
- Positive prediction value on nucleotide level: nPPV = nTP/(nTP+nFP);
- Specificity on nucleotide level: nSP = nTN/(nTN+nFP)
- Performance coefficient on nucleotide level: nPC = nTP/(nTP+nFN +nFP);
- Correlated co efficient on nucleotide level:

$$nCC = \frac{nTP * nTN \quad nFN * nFP}{\sqrt{(nTP + nFN)(nTN + nFP)}(nTP + nFP)(nTN + nFN)}$$

- Sensitivity on site level: sSN = sTP/(sTP+sFN);
- Positive prediction value on site level: sPPV = sTP/(sTP+sFP);
- Average site performance on site level: sASP = (sSN+sPPV)/2;
- We add another widely used statistic F-score on site level as following:

$$FS = \frac{2 * sSN * sPPV}{sSN + sPPV}$$

The values of these statistics on top one and top five prediction of MP^3 and other seven tools are shown in Table S2.

Result S1: Analysis of Sigma 70 binding on *E. coli* promoter sequences.

We conducted an analysis to see the correlation between orthology and Sigma 70 binding. The Sigma 70 binding information was downloaded from the RegulonDB database. All the experimentally confirmed, strongly validated and weakly validated binding activities are included in this analysis. For each group of orthologous promoter sequences in *E. coli*, the ratio of sequences with Sigma 70 binding to the total number in this group was calculated and was shown in Fig. S3. We found that the promoters with more orthologs tend to have a higher ratio, indicating a more enriched Sigma70 motif enrichment. In this figure, we also find the sigma 70 motif enrichment are flexible in some regions, for which we have not found a reasonable explanation. We believe that the evolution of regulation is a complicated progress and driven by multiple factors and future work integrating the ever increasing Omics data may provide new clues.

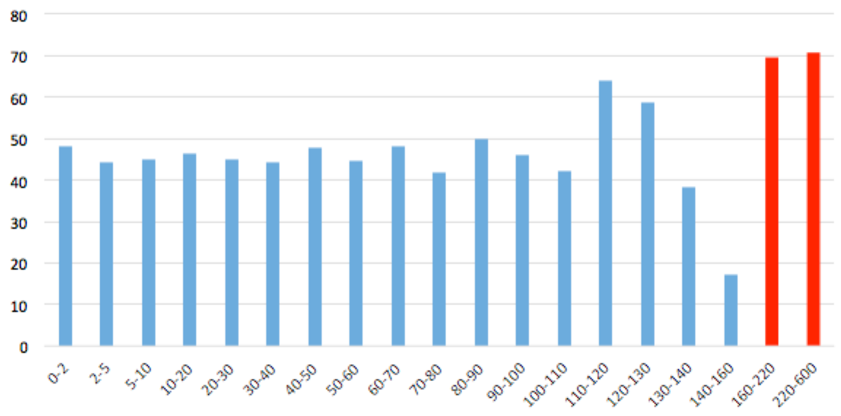

Fig. S3. Sigma70 motif enrichment analysis. The *x*-axis is the interval of orthologous promoters; the *y*-axis is the percentage of promoter sequences with known Sigma 70 binding in the corresponding interval.

Table S2. A: Top one prediction

Tools\Scores	nSN	nPPV	nSP	nPC	nCC	sSN	sPPV	sFscore	sASP
**Bioprospector**	0.065	0.293	0.968	0.056	0.065	0.119	0.388	0.182	0.254
**BOBRO**	0.055	0.308	0.975	0.049	0.066	0.112	0.43	0.178	0.271
**CONSENSUS**	0.056	0.286	0.972	0.049	0.058	0.099	0.371	0.156	0.235
**CUBIC**	0.06	0.309	0.973	0.053	0.069	0.109	0.402	0.171	0.255
**MDscan**	0.068	0.326	0.971	0.06	0.081	0.124	0.421	0.191	0.272
**MEME**	0.024	0.162	0.975	0.021	0	0.046	0.235	0.077	0.14
**MFP**	0.015	0.302	0.993	0.015	0.033	0.031	0.391	0.057	0.211
**MP3-CBR**	0.167	0.379	0.945	0.131	0.16	0.222	0.607	0.325	0.415
**MP3**	0.147	0.385	0.953	0.119	0.152	0.208	0.584	0.306	0.396

B: Top five predictions

Tools\Scores	nSN	nPPV	nSP	nPC	nCC	sSN	sPPV	sFscore	sASP
**Bioprospector**	0.14	0.248	0.914	0.099	0.07	0.231	0.198	0.213	0.215
**BOBRO**	0.197	0.268	0.891	0.128	0.1	0.333	0.315	0.324	0.324
**CONSENSUS**	0.096	0.239	0.938	0.074	0.051	0.16	0.156	0.158	0.158
**CUBIC**	0.233	0.283	0.881	0.146	0.123	0.373	0.312	0.339	0.342
**MDscan**	0.15	0.254	0.911	0.104	0.076	0.239	0.212	0.225	0.226
**MEME**	0.13	0.178	0.879	0.081	0.01	0.237	0.245	0.241	0.241
**MFP**	0.054	0.256	0.968	0.047	0.045	0.096	0.278	0.142	0.187
**MP3-CBR**	0.483	0.243	0.696	0.193	0.142	0.589	0.414	0.486	0.501
**MP3**	0.414	0.248	0.746	0.183	0.133	0.553	0.394	0.46	0.474

Result S2. MP^3 Implement in DMINDA: an application example

To facilitate the usage of MP^3, we have implemented all the functions of MP^3 in the integrated motif identification and analyses web server, DMINDA [8]. We listed all genes for 2,072 prokaryotic genomes and collected the orthologous promoter of them as did on *E. coli*, thus the users can perform motif detection by several clicks. We use the gene *argR* as an example to show how our server works. The gene *argR* composes a single gene operon [9]. Its corresponding protein *ArgR* plays an important role in repressing the transcription of several genes involved in biosynthesis and transport of arginine, transport of histidine, and its own synthesis [10] and activating genes for arginine catabolism [11, 12].

Step 1: Go to the main page of DMINDA (http://csbl.bmb.uga.edu/DMINDA/), and click on the MP^3 logo in the middle area (Fig. S4A). A start page will provide two options for users to select interested genes in list or upload promoter sequences data if available. Actually, MP^3 provides a list including 2,072 organisms will pop out with the following menus: (i) Species, (ii) NCs, (iii) Genes, (iv) Operons and (v) Statistics. For *argR* in *E.coli*, users can select it in the list as the following steps.

Step 2: To prepare the reference promoter sequences, users can search for 'NC_000913' or '*Escherichia coli* K-12 MG1655' in the organism table. Click on 'NC_000913', and a table of operons for this genome will be shown along with a button 'Get promoters'. Search for the gene name, '*argR*' or 'b3237', in the operon table and check its box, and then click on 'Get promoters' to get the corresponding orthologous promoters. The sequences will show in a text area for mortification if needed or upload by or directly click "Upload promoters" button.

Step 3: Now click "Submit" to run the MP^3 prediction job. Here the user has the option to enter an email address for results retrieval if preferred.

For this example, MP^3 can finish motif finding within 10 minutes, and entering the job ID 2015092045241m into the searching box on our server can retrieve the prediction results. A result page lists the curve representing the voting scores along with several CBRs and corresponding Motif Profiles for the given query sequences (Fig. S4BC). The right peak in the figure successfully covered two documented TF binding sites located at -62 and -42 upstream regions of the gene argR, and the weblog of the first output motif profile coincides with the motif profiles provided by RegulonDB (Fig. S5). All the motif profiles are listed in a table, with each row representing one motif showing the following information: motif logo, width, *p*-value, the number of instances, the corresponding CBRs, the genomic location for each identified instance in the query sequences, the sequence alignment of the motif profile, and a clickable link to the position weight matrix, position-specific scoring matrix and a graphical mapping of predicted instances in the query sequences of the motif (Fig. S4D). The input sequence data and the plain text for prediction are also provided (Fig. S4C&E). Users can also choose the predicted motifs to do further analysis by function provided by DMINDA (Fig.

S4C)

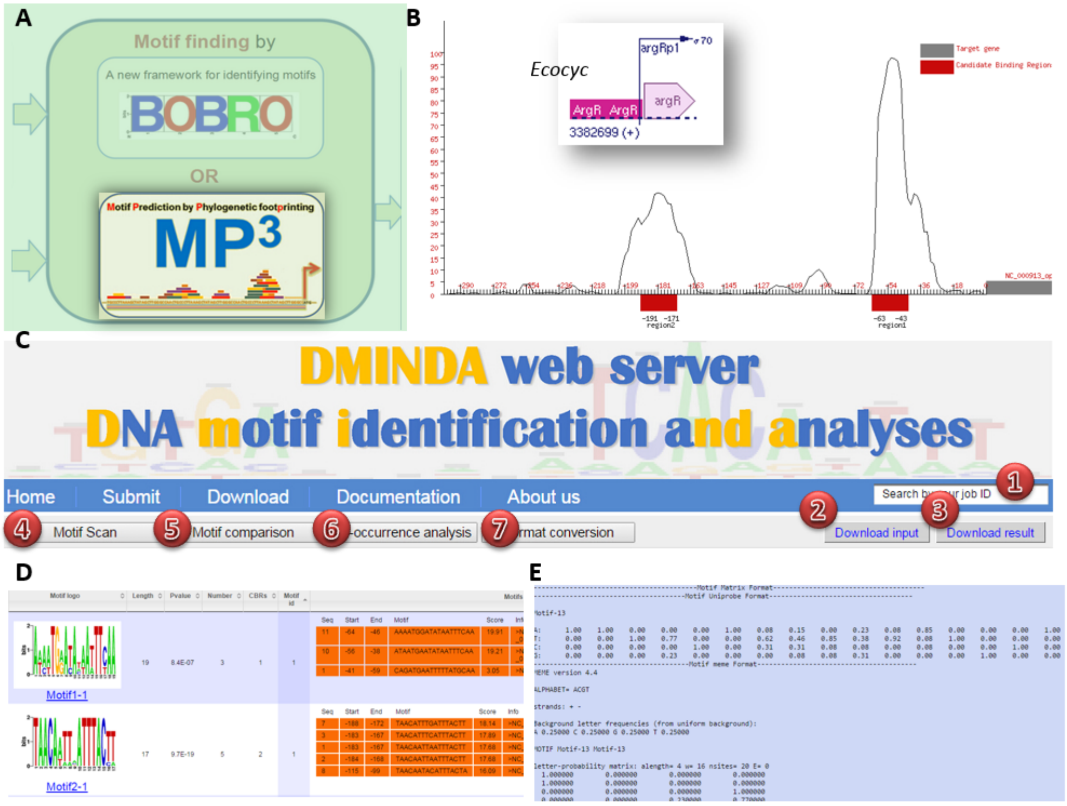

Fig. S4: Motif finding for *argR* using MP^3. (A) MP^3 entry on DMINDA. (B) Voting score curve along with three CBRs. (C) Job accessing box and functional buttons for data acquiring and further analysis of predicted motifs, where (1) is a searching box showing corresponding job ID and users can download the submitted query and the predictions by clicking (2) and (3) respectively; The buttons (4), (5) and (6) allow users to do three follow-up motif analysis functions and (7) provides a format conversion capability to inter-convert file formats used in our server, MEME and the Uniprobe database. (D) The information of a motif profile, including motif logo, width, and details of sequence alignment, also the location information of the predicted motif instances compared to downstream genes. (E) the detailed information of predication, including consensus, PWM, PSSM, information content and results in other formats, e.g. MEME and Uniprobe. The sketch about *argR* regulation in B is from EcoCyc.

Table S3: the statistics of MP^3-CMP on Near and Far promoter regions.

Top1	nSN	nPPV	nSP	nPC	nCC	sSN	sPPV	sFscore	sASP
**Near**	0.201	0.418	0.932	0.157	0.181	0.274	0.631	0.382	0.453
**Far**	0.105	0.343	0.964	0.088	0.118	0.147	0.518	0.229	0.333
**Top5**	**nSN**	**nPPV**	**nSP**	**nPC**	**nCC**	**sSN**	**sPPV**	**sFscore**	**sASP**
**Near**	0.475	0.278	0.701	0.213	0.148	0.631	0.447	0.524	0.539
**Far**	0.368	0.222	0.773	0.161	0.116	0.482	0.346	0.403	0.414

Fig. S5

ArgR TFBSs in E.coli	
CAAATGAATAATCATCCAT	GTATTCACTTTATATGCAC
ATTGTGAATTAATATGCAA	TTAATGAAAAATAATACGT
AAAGTGAGTGAATATTCTC	GAAATGCATACTTAAGCAT
AAAGTGAATTTTAATTCAA	CATGTGAATGAATATCCAG
CAAATGAATAATTACACAT	TGATTAAATGAAAACTCAT
TTATTGCATATAAATTCAC	ATTTTGCATAAAAATTCAG
TTAATCATGTTTATTGCAT	TGTTTGCATAAAAATTCAT
ATATTGCATAATTATTCTG	TGTATGCACAATAATGTTG
CTTTTAACTTTTAAAGCAG	TTTGTGGTTATAATTTCAC
TTATGCACTTTTATCACTG	TTAGTGATTTTTTATGCAT
GGTTTGGTTATAACTCCTT	AGTATCAATATTCATGCAG
ATATGCACTTTAAATGCAT	TTTATGAATAAAAATACAC
	AAATTGAATTTTAATTCAT

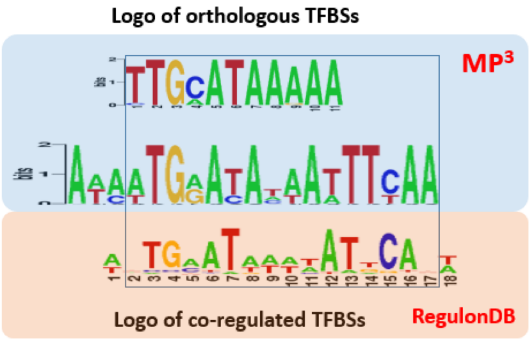

Fig. S5. The ArgR motif profiles from co-regulatory genes and orthologues genes. The left two volumes are the known ArgR binding sites in E.coli genome. The eight binding sites with underline are those who show high similarity with motif profiles from orthologous genes. The right figure shows the alignment of two motif profiles from MP^3 and one motif profile from co-regulatory genes by RegulonDB.

Additional References

1. Blanchette, M. and M. Tompa, *Discovery of regulatory elements by a computational method for phylogenetic footprinting.* Genome Res, 2002. **12**(5): p. 739-48.
2. Manson McGuire, A. and G.M. Church, *Predicting regulons and their cis-regulatory motifs by comparative genomics.* Nucleic Acids Res, 2000. **28**(22): p. 4523-30.
3. McCue, L.A., et al., *Factors influencing the identification of transcription factor binding sites by cross-species comparison.* Genome Res, 2002. **12**(10): p. 1523-32.
4. McCue, L., et al., *Phylogenetic footprinting of transcription factor binding sites in proteobacterial genomes.* Nucleic Acids Res, 2001. **29**(3): p. 774-82.
5. Thompson, J.D., D.G. Higgins, and T.J. Gibson, *CLUSTAL W: improving the sensitivity of progressive multiple sequence alignment through sequence weighting, position-specific gap penalties and weight matrix choice.* Nucleic Acids Res, 1994. **22**(22): p. 4673-80.
6. Li, G., et al., *Integration of sequence-similarity and functional association information can overcome intrinsic problems in orthology mapping across bacterial genomes.* Nucleic Acids Res, 2011. **39**(22): p. e150.
7. Tompa, M., et al., *Assessing computational tools for the discovery of transcription factor binding sites.* Nat Biotechnol, 2005. **23**(1): p. 137-44.
8. Ma, Q., et al., *DMINDA: an integrated web server for DNA motif identification and analyses.* Nucleic Acids Res, 2014. **42**(Web Server issue): p. W12-9.
9. Gama-Castro, S., et al., *RegulonDB (version 6.0): gene regulation model of Escherichia coli K-12 beyond transcription, active (experimental) annotated promoters and Textpresso navigation.* Nucleic Acids Res, 2008. **36**(Database issue): p. D120-4.
10. Caldara, M., D. Charlier, and R. Cunin, *The arginine regulon of Escherichia coli: whole-system transcriptome analysis discovers new genes and provides an integrated view of arginine regulation.* Microbiology, 2006. **152**(Pt 11): p. 3343-54.
11. Kiupakis, A.K. and L. Reitzer, *ArgR-independent induction and ArgR-dependent superinduction of the astCADBE operon in Escherichia coli.* J Bacteriol, 2002. **184**(11): p. 2940-50.
12. Keseler, I.M., et al., *EcoCyc: a comprehensive database resource for Escherichia coli.* Nucleic Acids Res, 2005. **33**(Database issue): p. D334-7.

Table S1

Annotation Cluster 1	Enrichment Score: 4.41108219357495											
Category	Term	Count	%	PValue	Genes	List Total	Pop Hits	Pop Total	Fold Enrichment	Bonferroni	Benjamini	FDR
SP_PIR_KEYWORDS	protein biosynthesis	9	9	1.75E-09	5260574, 5282105, 5301931, 5287199, 5275125, 5277581, 5262183, 5308036, 5271238	100	159	47487	26.87943	3.17E-07	3.17E-07	2.16E-06
SP_PIR_KEYWORDS	ribosomal protein	7	7	1.87E-08	5260574, 5282105, 5301931, 5277581, 5269382, 5262183, 5308036	100	80	47487	41.55113	3.38E-06	1.69E-06	2.30E-05
SP_PIR_KEYWORDS	ribosome	6	6	1.25E-07	5260574, 5282105, 5301931, 5277581, 5262183, 5308036	100	56	47487	50.87893	2.26E-05	4.52E-06	1.54E-04
GOTERM_MF_FAT	GO:0005198~structural molecule activity	9	9	1.53E-07	5307068, 5260574, 5282105, 5301931, 5277618, 5257233, 5277581, 5262183, 5308036	73	149	17785	14.71591	2.83E-05	2.83E-05	1.89E-04
SP_PIR_KEYWORDS	ribonucleoprotein	6	6	5.84E-07	5260574, 5282105, 5301931, 5277581, 5262183, 5308036	100	76	47487	37.48974	1.06E-04	1.51E-05	7.20E-04
GOTERM_BP_FAT	GO:0006412~translation	10	10	4.97E-06	5260574, 5282105, 5301931, 5287199, 5275125, 5277581, 5279415, 5262183, 5308036, 5271238	85	253	16709	7.769821	0.001752	0.001752	0.006804
GOTERM_CC_FAT	GO:0005840~ribosome	7	7	6.91E-06	5260574, 5282105, 5301931, 5277581, 5269382, 5262183, 5308036	43	83	7281	14.28047	3.18E-04	3.18E-04	0.006531
GOTERM_CC_FAT	GO:0030529~ribonucleoprotein complex	7	7	1.04E-05	5260574, 5282105, 5301931, 5277581, 5269382, 5262183, 5308036	43	89	7281	13.31774	4.77E-04	2.38E-04	0.0098
GOTERM_CC_FAT	GO:0043228~non-membrane-bounded organelle	10	10	1.39E-05	5307068, 5260574, 5282105, 5301931, 5257233, 5277581, 5284427, 5269382, 5262183, 5308036	43	262	7281	6.462808	6.37E-04	2.12E-04	0.013099
GOTERM_CC_FAT	GO:0043232~intracellular non-membrane-bounded organelle	10	10	1.39E-05	5307068, 5260574, 5282105, 5301931, 5257233, 5277581, 5284427, 5269382, 5262183, 5308036	43	262	7281	6.462808	6.37E-04	2.12E-04	0.013099
GOTERM_MF_FAT	GO:0003735~structural constituent of ribosome	6	6	1.49E-05	5260574, 5282105, 5301931, 5277581, 5262183, 5308036	73	77	17785	18.98417	0.002749	0.001375	0.018409
KEGG_PATHWAY	eck03010:Ribosome	6	6	3.43E-05	5260574, 5282105, 5301931, 5277581, 5262183, 5308036	51	54	7107	15.48366	0.024439	0.024439	0.051882
KEGG_PATHWAY	ecq03010:Ribosome	6	6	3.43E-05	5260574, 5282105, 5301931, 5277581, 5262183, 5308036	51	54	7107	15.48366	0.024439	0.024439	0.051882
KEGG_PATHWAY	ecz03010:Ribosome	6	6	3.43E-05	5260574, 5282105, 5301931, 5277581, 5262183, 5308036	51	54	7107	15.48366	0.024439	0.024439	0.051882
KEGG_PATHWAY	eum03010:Ribosome	6	6	3.43E-05	5260574, 5282105, 5301931, 5277581, 5262183, 5308036	51	54	7107	15.48366	0.024439	0.024439	0.051882
KEGG_PATHWAY	ecr03010:Ribosome	6	6	3.43E-05	5260574, 5282105, 5301931, 5277581,	51	54	7107	15.48366	0.024439	0.024439	0.051882

					5262183, 5308036							
KEGG_PATHWAY	ecw03010: Ribosome	6	6	3.43E-05	5260574, 5282105, 5301931, 5277581, 5262183, 5308036	51	54	7107	15.48366	0.024439	0.024439	0.051882
KEGG_PATHWAY	ect03010:Ribosome	6	6	3.75E-05	5260574, 5282105, 5301931, 5277581, 5262183, 5308036	51	55	7107	15.20214	0.026711	0.013446	0.056768
KEGG_PATHWAY	eci03010:Ribosome	6	6	3.75E-05	5260574, 5282105, 5301931, 5277581, 5262183, 5308036	51	55	7107	15.20214	0.026711	0.013446	0.056768
KEGG_PATHWAY	ecm03010: Ribosome	6	6	4.10E-05	5260574, 5282105, 5301931, 5277581, 5262183, 5308036	51	56	7107	14.93067	0.029138	0.009809	0.062003
KEGG_PATHWAY	ecl03010:Ribosome	6	6	4.10E-05	5260574, 5282105, 5301931, 5277581, 5262183, 5308036	51	56	7107	14.93067	0.029138	0.009809	0.062003
KEGG_PATHWAY	ecf03010:Ribosome	6	6	4.10E-05	5260574, 5282105, 5301931, 5277581, 5262183, 5308036	51	56	7107	14.93067	0.029138	0.009809	0.062003
KEGG_PATHWAY	ecx03010: Ribosome	6	6	4.10E-05	5260574, 5282105, 5301931, 5277581, 5262183, 5308036	51	56	7107	14.93067	0.029138	0.009809	0.062003
KEGG_PATHWAY	ecy03010: Ribosome	6	6	4.10E-05	5260574, 5282105, 5301931, 5277581, 5262183, 5308036	51	56	7107	14.93067	0.029138	0.009809	0.062003
KEGG_PATHWAY	ecg03010: Ribosome	6	6	4.10E-05	5260574, 5282105, 5301931, 5277581, 5262183, 5308036	51	56	7107	14.93067	0.029138	0.009809	0.062003
KEGG_PATHWAY	ecj03010:Ribosome	6	6	4.47E-05	5260574, 5282105, 5301931, 5277581, 5262183, 5308036	51	57	7107	14.66873	0.03173	0.008029	0.067605
KEGG_PATHWAY	ecv03010: Ribosome	5	5	1.08E-04	5260574, 5282105, 5301931, 5277581, 5262183	51	36	7107	19.35458	0.075147	0.015503	0.163715
KEGG_PATHWAY	ecc03010: Ribosome	6	6	1.47E-04	5260574, 5282105, 5301931, 5277581, 5262183, 5308036	51	73	7107	11.45367	0.100845	0.017561	0.222703
KEGG_PATHWAY	ece03010: Ribosome	6	6	2.02E-04	5260574, 5282105, 5301931, 5277581, 5262183, 5308036	51	78	7107	10.71946	0.135424	0.020573	0.304738
KEGG_PATHWAY	ecs03010: Ribosome	6	6	2.02E-04	5260574, 5282105, 5301931, 5277581, 5262183, 5308036	51	78	7107	10.71946	0.135424	0.020573	0.304738
KEGG_PATHWAY	eco03010: Ribosome	6	6	2.14E-04	5260574, 5282105, 5301931, 5277581, 5262183, 5308036	51	79	7107	10.58377	0.143184	0.019131	0.323587
KEGG_PATHWAY	ecd03010: Ribosome	5	5	5.29E-04	5260574, 5282105, 5277581, 5262183, 5308036	51	54	7107	12.90305	0.317652	0.041579	0.798446
KEGG_PATHWAY	ecp03010: Ribosome	5	5	5.68E-04	5260574, 5282105, 5301931, 5277581, 5262183	51	55	7107	12.66845	0.336401	0.040178	0.856399
SP_PIR_KEYWORDS	rna-binding	5	5	6.42E-04	5285358, 5260574, 5301931, 5266128, 5277581	100	186	47487	12.76532	0.109699	0.005518	0.788351
SP_PIR_KEYWORDS	rrna-binding	3	3	0.005133	5260574, 5301931, 5277581	100	51	47487	27.93353	0.605997	0.027022	6.147186
GOTERM_CC_FAT	GO:0033279~ribosomal subunit	3	3	0.00522	5301931, 5277581, 5262183	43	19	7281	26.73562	0.213968	0.029646	4.826592
GOTERM_MF_FAT	GO:0000049~tRNA binding	3	3	0.006114	5260574, 5301931, 5262183	73	29	17785	25.20312	0.678447	0.149631	7.307987
GOTERM_MF_FAT	GO:0003723~RNA binding	7	7	0.006579	5285358, 5260574, 5301931, 5266128, 5277581, 5262183, 5258016	73	414	17785	4.11935	0.705132	0.126888	7.843544
GOTERM_MF_FAT	GO:0019843~rRNA binding	3	3	0.018134	5260574, 5301931, 5277581	73	51	17785	14.33118	0.966141	0.229278	20.26364
Annotation Cluster	Enrichment Score: 2.984642764959887											

2												
Category	Term	Count	%	PValue	Genes	List Total	Pop Hits	Pop Total	Fold Enrichment	Bonferroni	Benjamini	FDR
SP_PIR_KEYWORDS	metal-binding	16	16	8.34E-07	5271647, 5305555, 5283477, 5302805, 5275125, 5275908, 5271238, 5283993, 5261315, 5307481, 5274886, 5279158, 5288791, 5257873, 5301118, 5287917	100	1558	47487	4.876714	1.51E-04	1.89E-05	0.001028
SP_PIR_KEYWORDS	magnesium	9	9	3.36E-06	5274010, 5305555, 5274886, 5302805, 5260139, 5271238, 5284352, 5301118, 5287917	100	427	47487	10.00897	6.07E-04	5.52E-05	0.004138
GOTERM_MF_FAT	GO:0043169~cation binding	23	23	0.001699	5271647, 5305555, 5283477, 5302805, 5275125, 5275908, 5281198, 5284352, 5271238, 5283993, 5274010, 5261315, 5285141, 5307481, 5274886, 5260139, 5296877, 5279158, 5288791, 5257873, 5301118, 5287917, 5291861	73	2876	17785	1.948363	0.269876	0.075623	2.08185
GOTERM_MF_FAT	GO:0000287~magnesium ion binding	9	9	0.001811	5274010, 5305555, 5274886, 5302805, 5260139, 5271238, 5284352, 5301118, 5287917	73	557	17785	3.936573	0.28492	0.064872	2.218113
GOTERM_MF_FAT	GO:0043167~ion binding	23	23	0.001819	5271647, 5305555, 5283477, 5302805, 5275125, 5275908, 5281198, 5284352, 5271238, 5283993, 5274010, 5261315, 5285141, 5307481, 5274886, 5260139, 5296877, 5279158, 5288791, 5257873, 5301118, 5287917, 5291861	73	2891	17785	1.938254	0.285979	0.054594	2.2278
SP_PIR_KEYWORDS	zinc	6	6	0.004171	5283993, 5271647, 5305555, 5283477, 5257873, 5301118	100	504	47487	5.653214	0.530723	0.023365	5.022814
GOTERM_MF_FAT	GO:0046872~metal ion binding	21	21	0.006339	5271647, 5305555, 5283477, 5302805, 5275125, 5275908, 5284352, 5271238, 5283993, 5274010, 5261315, 5285141, 5307481, 5274886, 5260139, 5279158, 5288791, 5257873, 5301118, 5287917, 5291861	73	2793	17785	1.831806	0.691638	0.136758	7.567315
GOTERM_MF_FAT	GO:0046914~transition metal ion binding	16	16	0.017686	5271647, 5305555, 5283477, 5275125, 5275908, 5283993, 5261315, 5285141, 5307481, 5260139, 5279158, 5288791, 5257873, 5301118, 5287917, 5291861	73	2075	17785	1.878594	0.963161	0.240503	19.81242
GOTERM_MF_FAT	GO:0008270~zinc ion binding	6	6	0.187617	5283993, 5271647, 5305555, 5283477, 5257873, 5301118	73	751	17785	1.946446	1	0.853685	92.35465
Annotation Cluster	Enrichment Score: 2.598447021863862											

3											
Category	Term	Count	%	PValue	Genes	List Total	Pop Hits	Pop Total	Fold Enrichment	Bonferroni	Benjamini	FDR
SP_PIR_KEYWORDS	transmembrane protein	11	11	2.38E-08	5280453, 5283429, 5262424, 5270183, 5260139, 5295417, 5286820, 5281198, 5304681, 5291861, 5275617	100	427	47487	12.23319	4.30E-06	1.43E-06	2.93E-05
SP_PIR_KEYWORDS	cell inner membrane	18	18	1.07E-07	5280453, 5305802, 5270183, 5284427, 5290163, 5275617, 5284923, 5283429, 5296702, 5260139, 5295417, 5286820, 5261931, 5269304, 5302970, 5300277, 5278591, 5291861	100	1732	47487	4.935139	1.95E-05	4.86E-06	1.32E-04
SP_PIR_KEYWORDS	cell membrane	22	22	1.16E-06	5280453, 5305802, 5270183, 5284427, 5290163, 5275617, 5284923, 5274010, 5283429, 5296702, 5260139, 5300359, 5295417, 5286820, 5261931, 5269304, 5302970, 5300277, 5280934, 5278591, 5304681, 5291861	100	3067	47487	3.406306	2.09E-04	2.09E-05	0.001425
SP_PIR_KEYWORDS	membrane	22	22	5.03E-06	5280453, 5305802, 5270183, 5284427, 5290163, 5275617, 5284923, 5274010, 5283429, 5296702, 5260139, 5300359, 5295417, 5286820, 5261931, 5269304, 5302970, 5300277, 5280934, 5278591, 5304681, 5291861	100	3367	47487	3.102804	9.10E-04	7.00E-05	0.006198
GOTERM_CC_FAT	GO:0009274~peptidoglycan-based cell wall	15	15	5.51E-04	5280453, 5270183, 5278413, 5297155, 5304594, 5275617, 5283429, 5260139, 5300359, 5295417, 5286820, 5261931, 5269304, 5278591, 5291861	43	946	7281	2.684867	0.025013	0.006313	0.519132
GOTERM_CC_FAT	GO:0005618~cell wall	15	15	6.47E-04	5280453, 5270183, 5278413, 5297155, 5304594, 5275617, 5283429, 5260139, 5300359, 5295417, 5286820, 5261931, 5269304, 5278591, 5291861	43	961	7281	2.642959	0.02934	0.005938	0.610007
GOTERM_CC_FAT	GO:0031967~organelle envelope	11	11	0.001122	5305802, 5283429, 5302495, 5270183, 5300359, 5295417, 5261931, 5269304, 5278591, 5291861, 5275617	43	566	7281	3.29078	0.050313	0.008567	1.055111
GOTERM_CC_FAT	GO:0019866~organelle inner membrane	11	11	0.001122	5305802, 5283429, 5302495, 5270183, 5300359, 5295417, 5261931, 5269304, 5278591, 5291861, 5275617	43	566	7281	3.29078	0.050313	0.008567	1.055111
GOTERM_CC_FAT	GO:0031090~organelle membrane	11	11	0.001353	5305802, 5283429, 5302495, 5270183, 5300359, 5295417, 5261931, 5269304, 5278591, 5291861, 5275617	43	580	7281	3.211347	0.060371	0.008856	1.27134

SP_PIR_KEYWORDS	transmembrane	16	16	0.002167	5280453, 5305802, 5270183, 5290163, 5275617, 5284923, 5283429, 5296702, 5260139, 5295417, 5286820, 5261931, 5302970, 5278591, 5304681, 5291861	100	3144	47487	2.416641	0.324775	0.014991	2.639497
GOTERM_CC_FAT	GO:0030312~external encapsulating structure	16	16	0.077945	5280453, 5270183, 5278413, 5297155, 5304594, 5275617, 5283429, 5260139, 5300359, 5295417, 5286820, 5261931, 5269304, 5278591, 5274831, 5291861	43	1814	7281	1.4935	0.976077	0.234049	53.56039
GOTERM_CC_FAT	GO:0005886~plasma membrane	22	22	0.148453	5280453, 5305802, 5270183, 5284427, 5290163, 5275617, 5284923, 5274010, 5283429, 5296702, 5260139, 5300359, 5295417, 5286820, 5261931, 5269304, 5302970, 5300277, 5280934, 5278591, 5304681, 5291861	43	2979	7281	1.250474	0.999384	0.389097	78.1051
GOTERM_CC_FAT	GO:0031975~envelope	14	14	0.169804	5305802, 5280453, 5270183, 5275617, 5283429, 5260139, 5302495, 5300359, 5295417, 5261931, 5269304, 5278591, 5274831, 5291861	43	1715	7281	1.38225	0.999808	0.395616	82.77684
UP_SEQ_FEATURE	topological domain:Periplasmic	10	10	0.714548	5284923, 5305802, 5280453, 5260139, 5295417, 5261931, 5302970, 5290163, 5278591, 5291861	100	985	9468	0.961218	1	1	99.99998
UP_SEQ_FEATURE	topological domain:Cytoplasmic	10	10	0.714548	5284923, 5305802, 5280453, 5260139, 5295417, 5261931, 5302970, 5290163, 5278591, 5291861	100	985	9468	0.961218	1	1	99.99998
UP_SEQ_FEATURE	transmembrane region	16	16	0.864853	5280453, 5305802, 5270183, 5290163, 5275617, 5284923, 5283429, 5296702, 5260139, 5295417, 5286820, 5261931, 5302970, 5278591, 5304681, 5291861	100	1793	9468	0.844886	1	1	100
GOTERM_CC_FAT	GO:0031224~intrinsic to membrane	17	17	0.99909	5280453, 5305802, 5270183, 5290163, 5275617, 5284923, 5283429, 5296702, 5260139, 5295417, 5286820, 5261931, 5302970, 5280934, 5278591, 5304681, 5291861	43	4417	7281	0.651695	1	1	100
GOTERM_CC_FAT	GO:0016021~integral to membrane	16	16	0.999204	5280453, 5305802, 5270183, 5290163, 5275617, 5284923, 5283429, 5296702, 5260139, 5295417, 5286820, 5261931, 5302970, 5278591, 5304681, 5291861	43	4269	7281	0.634624	1	0.999999	100
Annotation Cluster 4	Enrichment Score: 2.4359129505447723											
Category	Term	Count	%	PValue	Genes	List Total	Pop Hits	Pop Total	Fold Enrich	Bonferroni	Benjamini	FDR

									ment			
GOTERM_BP_FAT	GO:0044271~nitrogen compound biosynthetic process	17	17	1.95E-04	5291640, 5289443, 5266336, 5286049, 5302805, 5276530, 5297155, 5279860, 5284352, 5308498, 5261315, 5283429, 5274886, 5284767, 5288791, 5296304, 5300523	85	1164	16709	2.870962	0.066659	0.033904	0.267259
SP_PIR_KEYWORDS	amino-acid biosynthesis	6	6	3.45E-04	5308498, 5291640, 5266336, 5302805, 5284352, 5300523	100	286	47487	9.962308	0.060517	0.00328	0.424311
GOTERM_BP_FAT	GO:0046394~carboxylic acid biosynthetic process	10	10	0.007373	5308498, 5291640, 5266336, 5274886, 5286049, 5302805, 5288791, 5274212, 5284352, 5300523	85	685	16709	2.869729	0.926627	0.229888	9.637103
GOTERM_BP_FAT	GO:0016053~organic acid biosynthetic process	10	10	0.007578	5308498, 5291640, 5266336, 5274886, 5286049, 5302805, 5288791, 5274212, 5284352, 5300523	85	688	16709	2.857216	0.931783	0.216588	9.892189
GOTERM_BP_FAT	GO:0008652~cellular amino acid biosynthetic process	8	8	0.020633	5308498, 5291640, 5266336, 5274886, 5286049, 5302805, 5284352, 5300523	85	550	16709	2.859294	0.999364	0.32115	24.83704
GOTERM_BP_FAT	GO:0009309~amine biosynthetic process	8	8	0.031207	5308498, 5291640, 5266336, 5274886, 5286049, 5302805, 5284352, 5300523	85	600	16709	2.62102	0.999986	0.372693	35.21947
Annotation Cluster 5	Enrichment Score: 2.371816442285723											
Category	Term	Count	%	PValue	Genes	List Total	Pop Hits	Pop Total	Fold Enrichment	Bonferroni	Benjamini	FDR
SP_PIR_KEYWORDS	glycosyltransferase	7	7	6.01E-06	5302495, 5302805, 5260139, 5278574, 5296877, 5281198, 5296304	100	213	47487	15.60606	0.001087	7.77E-05	0.007407
SP_PIR_KEYWORDS	lipopolysaccharide biosynthesis	6	6	2.33E-05	5302495, 5260139, 5286820, 5278574, 5297155, 5292331	100	161	47487	17.69702	0.004205	2.48E-04	0.028697
GOTERM_MF_FAT	GO:0042280~cell surface antigen activity, host-interacting	5	5	1.31E-04	5302495, 5260139, 5286820, 5297155, 5276311	73	64	17785	19.0336	0.023868	0.00802	0.161448
GOTERM_BP_FAT	GO:0000271~polysaccharide biosynthetic process	9	9	0.005728	5302495, 5260139, 5286820, 5278574, 5296877, 5297155, 5304594, 5276311, 5292331	85	540	16709	3.276275	0.868358	0.201721	7.564645
GOTERM_BP_FAT	GO:0005976~polysaccharide metabolic process	10	10	0.009378	5302495, 5260139, 5286820, 5278574, 5296877, 5297155, 5304594, 5276311, 5281198, 5292331	85	712	16709	2.760905	0.964069	0.225748	12.10553
GOTERM_BP_FAT	GO:0016051~carbohydrate biosynthetic process	9	9	0.012283	5302495, 5260139, 5286820, 5278574, 5296877, 5297155, 5304594, 5276311, 5292331	85	617	16709	2.867404	0.987256	0.252368	15.5697
GOTERM_BP_FAT	GO:0033692~cellular polysaccharide biosyntheti	7	7	0.016445	5302495, 5260139, 5286820, 5278574, 5296877, 5297155, 5292331	85	406	16709	3.389249	0.99713	0.291296	20.31382

	c process											
GOTERM_BP_FAT	GO:0044264~cellular polysaccharide metabolic process	7	7	0.021167	5302495, 5260139, 5286820, 5278574, 5296877, 5297155, 5292331	85	430	16709	3.200082	0.999475	0.314499	25.39584
GOTERM_BP_FAT	GO:0009103~lipopolysaccharide biosynthetic process	6	6	0.021244	5302495, 5260139, 5286820, 5278574, 5297155, 5292331	85	315	16709	3.744314	0.999489	0.302989	25.47659
GOTERM_BP_FAT	GO:0008653~lipopolysaccharide metabolic process	6	6	0.023646	5302495, 5260139, 5286820, 5278574, 5297155, 5292331	85	324	16709	3.640305	0.999786	0.318849	27.9424
GOTERM_BP_FAT	GO:0034637~cellular carbohydrate biosynthetic process	7	7	0.034087	5302495, 5260139, 5286820, 5278574, 5296877, 5297155, 5292331	85	481	16709	2.86078	0.999995	0.375541	37.80746
GOTERM_BP_FAT	GO:0008610~lipid biosynthetic process	7	7	0.03933	5302495, 5260139, 5286820, 5278574, 5297155, 5274212, 5292331	85	498	16709	2.763123	0.999999	0.39701	42.2744
COG_ONTOLOGY	Cell envelope biogenesis, outer membrane	4	4	0.051929	5302495, 5270183, 5260139, 5292331	16	389	6729	4.32455	0.443774	0.443774	28.26486
Annotation Cluster 6	Enrichment Score: 2.0607700596491267											
Category	Term	Count	%	PValue	Genes	List Total	Pop Hits	Pop Total	Fold Enrichment	Bonferroni	Benjamini	FDR
KEGG_PATHWAY	ecv00290:Valine, leucine and isoleucine biosynthesis	3	3	0.008501	5287199, 5284352, 5300523	51	20	7107	20.90294	0.997896	0.336961	12.12709
KEGG_PATHWAY	ecm00290:Valine, leucine and isoleucine biosynthesis	3	3	0.008501	5287199, 5284352, 5300523	51	20	7107	20.90294	0.997896	0.336961	12.12709
KEGG_PATHWAY	ecf00290:Valine, leucine and isoleucine biosynthesis	3	3	0.008501	5287199, 5284352, 5300523	51	20	7107	20.90294	0.997896	0.336961	12.12709
KEGG_PATHWAY	eck00290:Valine, leucine and isoleucine biosynthesis	3	3	0.008501	5287199, 5284352, 5300523	51	20	7107	20.90294	0.997896	0.336961	12.12709
KEGG_PATHWAY	ect00290:Valine, leucine and isoleucine biosynthesis	3	3	0.008501	5287199, 5284352, 5300523	51	20	7107	20.90294	0.997896	0.336961	12.12709

KEGG_PATHWAY	ece00290:Valine, leucine and isoleucine biosynthesis	3	3	0.008501	5287199, 5284352, 5300523	51	20	7107	20.90294	0.997896	0.336961	12.12709
KEGG_PATHWAY	eci00290:Valine, leucine and isoleucine biosynthesis	3	3	0.008501	5287199, 5284352, 5300523	51	20	7107	20.90294	0.997896	0.336961	12.12709
KEGG_PATHWAY	ecc00290:Valine, leucine and isoleucine biosynthesis	3	3	0.008501	5287199, 5284352, 5300523	51	20	7107	20.90294	0.997896	0.336961	12.12709
KEGG_PATHWAY	ecr00290:Valine, leucine and isoleucine biosynthesis	3	3	0.008501	5287199, 5284352, 5300523	51	20	7107	20.90294	0.997896	0.336961	12.12709
KEGG_PATHWAY	ecq00290:Valine, leucine and isoleucine biosynthesis	3	3	0.008501	5287199, 5284352, 5300523	51	20	7107	20.90294	0.997896	0.336961	12.12709
KEGG_PATHWAY	ecg00290:Valine, leucine and isoleucine biosynthesis	3	3	0.008501	5287199, 5284352, 5300523	51	20	7107	20.90294	0.997896	0.336961	12.12709
KEGG_PATHWAY	ecx00290:Valine, leucine and isoleucine biosynthesis	3	3	0.008501	5287199, 5284352, 5300523	51	20	7107	20.90294	0.997896	0.336961	12.12709
KEGG_PATHWAY	ecw00290:Valine, leucine and isoleucine biosynthesis	3	3	0.008501	5287199, 5284352, 5300523	51	20	7107	20.90294	0.997896	0.336961	12.12709
KEGG_PATHWAY	ecz00290:Valine, leucine and isoleucine biosynthesis	3	3	0.009354	5287199, 5284352, 5300523	51	21	7107	19.90756	0.99887	0.345622	13.26476
KEGG_PATHWAY	ecj00290:Valine, leucine and isoleucine biosynthesis	3	3	0.009354	5287199, 5284352, 5300523	51	21	7107	19.90756	0.99887	0.345622	13.26476
KEGG_PATHWAY	eum00290:Valine, leucine and isoleucine	3	3	0.009354	5287199, 5284352, 5300523	51	21	7107	19.90756	0.99887	0.345622	13.26476

	biosynthesis											
KEGG_PATHWAY	eco00290: Valine, leucine and isoleucine biosynthesis	3	3	0.009354	5287199, 5284352, 5300523	51	21	7107	19.90756	0.99887	0.345622	13.26476
Annotation Cluster 7	Enrichment Score: 1.883568965152314											
Category	Term	Count	%	PValue	Genes	List Total	Pop Hits	Pop Total	Fold Enrichment	Bonferroni	Benjamini	FDR
SP_PIR_KEYWORDS	periplasmic space	4	4	1.17E-04	5305555, 5271782, 5300359, 5274831	100	45	47487	42.21067	0.02096	0.001176	0.144186
SP_PIR_KEYWORDS	periplasm	5	5	0.003459	5305555, 5278413, 5271782, 5257606, 5274831	100	295	47487	8.048644	0.4659	0.020028	4.182038
SP_PIR_KEYWORDS	signal	10	10	0.005117	5305555, 5278413, 5271782, 5300359, 5269304, 5284427, 5280934, 5257606, 5291861, 5274831	100	1549	47487	3.065655	0.604908	0.027748	6.129537
GOTERM_CC_FAT	GO:0042597~periplasmic space	7	7	0.186789	5307068, 5305555, 5278413, 5271782, 5257233, 5257606, 5274831	43	672	7281	1.763808	0.999926	0.410452	85.83365
UP_SEQ_FEATURE	signal peptide	10	10	0.98743	5305555, 5278413, 5271782, 5300359, 5269304, 5284427, 5280934, 5257606, 5291861, 5274831	100	1549	9468	0.611233	1	1	100
Annotation Cluster 8	Enrichment Score: 1.8719427113268834											
Category	Term	Count	%	PValue	Genes	List Total	Pop Hits	Pop Total	Fold Enrichment	Bonferroni	Benjamini	FDR
SP_PIR_KEYWORDS	cell cycle	4	4	0.002271	5284923, 5307240, 5304594, 5276311	100	124	47487	15.31839	0.33731	0.015123	2.763681
SP_PIR_KEYWORDS	cell division	4	4	0.006338	5284923, 5307240, 5304594, 5276311	100	179	47487	10.61162	0.683603	0.028359	7.5391
GOTERM_BP_FAT	GO:0007049~cell cycle	4	4	0.035382	5284923, 5307240, 5304594, 5276311	85	143	16709	5.498643	0.999997	0.375603	38.93958
GOTERM_BP_FAT	GO:0051301~cell division	4	4	0.063882	5284923, 5307240, 5304594, 5276311	85	182	16709	4.320362	1	0.476543	59.50536
Annotation Cluster 9	Enrichment Score: 1.7868084320340452											
Category	Term	Count	%	PValue	Genes	List Total	Pop Hits	Pop Total	Fold Enrichment	Bonferroni	Benjamini	FDR
KEGG_PATHWAY	ecd02020: Two-component system	6	6	0.001627	5305802, 5266336, 5261931, 5269304, 5300514, 5292331	51	123	7107	6.797704	0.691368	0.10136	2.435542
KEGG_PATHWAY	ecg02020: Two-component system	6	6	0.00181	5305802, 5266336, 5261931, 5269304, 5300514, 5292331	51	126	7107	6.635854	0.729658	0.103275	2.70622
KEGG_PATHWAY	ecj02020:Two-component system	6	6	0.002221	5305802, 5266336, 5261931, 5269304, 5300514, 5292331	51	132	7107	6.334225	0.799221	0.116182	3.311378
KEGG_PATHWAY	eco02020: Two-comp	6	6	0.002296	5305802, 5266336, 5261931, 5269304,	51	133	7107	6.286599	0.809779	0.111784	3.420868

	onent system				5300514, 5292331							
KEGG_PATHWAY	ect02020:Two-component system	5	5	0.010953	5305802, 5266336, 5261931, 5269304, 5300514	51	124	7107	5.61907	0.999648	0.373579	15.36076
KEGG_PATHWAY	ecr02020:Two-component system	5	5	0.010953	5305802, 5266336, 5261931, 5269304, 5300514	51	124	7107	5.61907	0.999648	0.373579	15.36076
KEGG_PATHWAY	ecq02020:Two-component system	5	5	0.012203	5305802, 5266336, 5261931, 5269304, 5300514	51	128	7107	5.443474	0.999859	0.388886	16.96615
KEGG_PATHWAY	ecf02020:Two-component system	5	5	0.012529	5305802, 5261931, 5269304, 5300514, 5292331	51	129	7107	5.401277	0.999889	0.380662	17.38044
KEGG_PATHWAY	ecz02020:Two-component system	5	5	0.012529	5305802, 5266336, 5261931, 5269304, 5300514	51	129	7107	5.401277	0.999889	0.380662	17.38044
KEGG_PATHWAY	eum02020:Two-component system	5	5	0.012529	5305802, 5266336, 5261931, 5269304, 5300514	51	129	7107	5.401277	0.999889	0.380662	17.38044
KEGG_PATHWAY	ecx02020:Two-component system	5	5	0.013541	5305802, 5261931, 5269304, 5300514, 5292331	51	132	7107	5.27852	0.999947	0.388703	18.65365
KEGG_PATHWAY	ecm02020:Two-component system	5	5	0.014604	5305802, 5261931, 5269304, 5300514, 5292331	51	135	7107	5.16122	0.999976	0.396983	19.9712
KEGG_PATHWAY	ecw02020:Two-component system	4	4	0.055071	5261931, 5269304, 5300514, 5292331	51	123	7107	4.531803	1	0.755924	57.58996
KEGG_PATHWAY	eck02020:Two-component system	4	4	0.058384	5305802, 5261931, 5269304, 5300514	51	126	7107	4.423903	1	0.76491	59.78653
KEGG_PATHWAY	ece02020:Two-component system	4	4	0.064111	5305802, 5266336, 5261931, 5300514	51	131	7107	4.255052	1	0.775744	63.33558
KEGG_PATHWAY	ecc02020:Two-component system	4	4	0.06767	5305802, 5266336, 5261931, 5300514	51	134	7107	4.159789	1	0.784114	65.39068
KEGG_PATHWAY	eci02020:Two-component system	3	3	0.232422	5305802, 5261931, 5300514	51	130	7107	3.215837	1	0.988221	98.17862
KEGG_PATHWAY	ecv02020:Two-component system	3	3	0.248106	5305802, 5261931, 5300514	51	136	7107	3.073962	1	0.990714	98.6676
Annotation Cluster 10	Enrichment Score: 1.7542380554284194											
Category	Term	Count	%	PValue	Genes	List Total	Pop Hits	Pop Total	Fold Enrichment	Bonferroni	Benjamini	FDR
SP_PIR_KEYWORDS	chemotaxis	3	3	0.001802	5305802, 5261931, 5284427	100	30	47487	47.487	0.27848	0.012971	2.198719
GOTERM_BP_FAT	GO:0007610~behavior	5	5	0.001932	5307068, 5305802, 5257233, 5261931, 5284427	85	105	16709	9.360784	0.494773	0.156914	2.613853
GOTERM_BP_FAT	GO:0007626~locomotory behavior	5	5	0.001932	5307068, 5305802, 5257233, 5261931, 5284427	85	105	16709	9.360784	0.494773	0.156914	2.613853

GOTERM_BP_FAT	GO:0042330~taxis	5	5	0.001932	5307068, 5305802, 5257233, 5261931, 5284427	85	105	16709	9.360784	0.494773	0.156914	2.613853
SP_PIR_KEYWORDS	flagellum	4	4	0.002769	5307068, 5257233, 5261931, 5284427	100	133	47487	14.2818	0.394585	0.017156	3.360545
GOTERM_BP_FAT	GO:0001539~ciliary or flagellar motility	4	4	0.002804	5307068, 5257233, 5261931, 5284427	85	56	16709	14.04118	0.628866	0.179826	3.772199
GOTERM_BP_FAT	GO:0048870~cell motility	4	4	0.002804	5307068, 5257233, 5261931, 5284427	85	56	16709	14.04118	0.628866	0.179826	3.772199
GOTERM_BP_FAT	GO:0051674~localization of cell	4	4	0.002804	5307068, 5257233, 5261931, 5284427	85	56	16709	14.04118	0.628866	0.179826	3.772199
GOTERM_BP_FAT	GO:0006928~cell motion	4	4	0.002949	5307068, 5257233, 5261931, 5284427	85	57	16709	13.79484	0.647452	0.138378	3.963804
GOTERM_CC_FAT	GO:0009425~flagellin-based flagellum basal body	3	3	0.00896	5307068, 5257233, 5284427	43	25	7281	20.31907	0.339016	0.044961	8.155345
GOTERM_MF_FAT	GO:0003774~motor activity	3	3	0.010872	5307068, 5257233, 5261931	73	39	17785	18.74078	0.867662	0.183101	12.6519
KEGG_PATHWAY	ecd02040:Flagellar assembly	3	3	0.02358	5307068, 5257233, 5261931	51	34	7107	12.29585	1	0.54302	30.32633
KEGG_PATHWAY	ect02040:Flagellar assembly	3	3	0.024898	5307068, 5257233, 5261931	51	35	7107	11.94454	1	0.546823	31.73713
KEGG_PATHWAY	ecj02040:Flagellar assembly	3	3	0.026246	5307068, 5257233, 5261931	51	36	7107	11.61275	1	0.550717	33.15196
KEGG_PATHWAY	ecr02040:Flagellar assembly	3	3	0.027622	5307068, 5257233, 5261931	51	37	7107	11.29889	1	0.554679	34.56901
KEGG_PATHWAY	ecg02040:Flagellar assembly	3	3	0.027622	5307068, 5257233, 5261931	51	37	7107	11.29889	1	0.554679	34.56901
KEGG_PATHWAY	eck02040:Flagellar assembly	3	3	0.029028	5307068, 5257233, 5261931	51	38	7107	11.00155	1	0.558692	35.98657
KEGG_PATHWAY	ecz02040:Flagellar assembly	3	3	0.029028	5307068, 5257233, 5261931	51	38	7107	11.00155	1	0.558692	35.98657
KEGG_PATHWAY	ecc02040:Flagellar assembly	3	3	0.029028	5307068, 5257233, 5261931	51	38	7107	11.00155	1	0.558692	35.98657
KEGG_PATHWAY	ecq02040:Flagellar assembly	3	3	0.029028	5307068, 5257233, 5261931	51	38	7107	11.00155	1	0.558692	35.98657
KEGG_PATHWAY	eco02040:Flagellar assembly	3	3	0.029028	5307068, 5257233, 5261931	51	38	7107	11.00155	1	0.558692	35.98657
KEGG_PATHWAY	ecv02040:Flagellar assembly	3	3	0.029028	5307068, 5257233, 5261931	51	38	7107	11.00155	1	0.558692	35.98657
KEGG_PATHWAY	ece02040:Flagellar assembly	3	3	0.029028	5307068, 5257233, 5261931	51	38	7107	11.00155	1	0.558692	35.98657
KEGG_PATHWAY	ecf02040:Flagellar assembly	3	3	0.029028	5307068, 5257233, 5261931	51	38	7107	11.00155	1	0.558692	35.98657
KEGG_PATHWAY	eci02040:Flagellar assembly	3	3	0.029028	5307068, 5257233, 5261931	51	38	7107	11.00155	1	0.558692	35.98657
KEGG_PATHWAY	ecx02040:Flagellar assembly	3	3	0.029028	5307068, 5257233, 5261931	51	38	7107	11.00155	1	0.558692	35.98657
KEGG_PATHWAY	ecw02040:Flagellar assembly	3	3	0.029028	5307068, 5257233, 5261931	51	38	7107	11.00155	1	0.558692	35.98657

GOTERM_CC_FAT	GO:0044460~flagellum part	3	3	0.037189	5307068, 5257233, 5284427	43	53	7281	9.584467	0.825059	0.159981	30.10696
GOTERM_CC_FAT	GO:0044461~flagellin-based flagellum part	3	3	0.037189	5307068, 5257233, 5284427	43	53	7281	9.584467	0.825059	0.159981	30.10696
GOTERM_CC_FAT	GO:0044463~cell projection part	3	3	0.037189	5307068, 5257233, 5284427	43	53	7281	9.584467	0.825059	0.159981	30.10696
GOTERM_BP_FAT	GO:0006935~chemotaxis	3	3	0.050897	5305802, 5261931, 5284427	85	72	16709	8.190686	1	0.437998	51.09814
GOTERM_CC_FAT	GO:0019861~flagellum	4	4	0.053983	5307068, 5257233, 5261931, 5284427	43	149	7281	4.545653	0.922133	0.191625	40.8163
KEGG_PATHWAY	ecm02040:Flagellar assembly	3	3	0.058728	5307068, 5257233, 5261931	51	56	7107	7.465336	1	0.755758	60.00848
KEGG_PATHWAY	eum02040:Flagellar assembly	3	3	0.058728	5307068, 5257233, 5261931	51	56	7107	7.465336	1	0.755758	60.00848
GOTERM_CC_FAT	GO:0009288~flagellin-based flagellum	3	3	0.061317	5307068, 5257233, 5284427	43	70	7281	7.256811	0.945565	0.200608	45.01359
GOTERM_CC_FAT	GO:0042995~cell projection	4	4	0.712735	5307068, 5257233, 5261931, 5284427	43	626	7281	1.081953	1	0.943239	99.99924
Annotation Cluster 11	Enrichment Score: 1.7345562736909936											
Category	Term	Count	%	PValue	Genes	List Total	Pop Hits	Pop Total	Fold Enrichment	Bonferroni	Benjamini	FDR
GOTERM_BP_FAT	GO:0018130~heterocycle biosynthetic process	9	9	0.001003	5261315, 5289443, 5266336, 5274886, 5286049, 5302805, 5284767, 5288791, 5296304	85	407	16709	4.3469	0.298309	0.111382	1.36491
GOTERM_BP_FAT	GO:0009110~vitamin biosynthetic process	7	7	0.002861	5289443, 5274886, 5286049, 5297155, 5288791, 5268716, 5287917	85	280	16709	4.914412	0.636309	0.155132	3.847802
GOTERM_BP_FAT	GO:0006766~vitamin metabolic process	7	7	0.004852	5289443, 5274886, 5286049, 5297155, 5288791, 5268716, 5287917	85	312	16709	4.41037	0.820381	0.193148	6.443566
GOTERM_BP_FAT	GO:0042364~water-soluble vitamin biosynthetic process	6	6	0.007722	5289443, 5274886, 5286049, 5297155, 5288791, 5268716	85	244	16709	4.833848	0.935192	0.203897	10.0712
GOTERM_BP_FAT	GO:0006767~water-soluble vitamin metabolic process	6	6	0.012694	5289443, 5274886, 5286049, 5297155, 5288791, 5268716	85	276	16709	4.273402	0.988998	0.245617	16.04981
GOTERM_BP_FAT	GO:0019438~aromatic compound biosynthetic process	5	5	0.019817	5261315, 5289443, 5266336, 5274886, 5286049	85	205	16709	4.794548	0.999146	0.324663	23.97495
GOTERM_BP_FAT	GO:0051188~cofactor biosynthetic process	7	7	0.02624	5261315, 5274886, 5286049, 5277618, 5284767, 5297155, 5287917	85	452	16709	3.044326	0.999916	0.335087	30.51978
GOTERM_BP_FAT	GO:0009108~coenzym	5	5	0.048755	5261315, 5274886, 5286049, 5297155,	85	273	16709	3.600302	1	0.434002	49.56473

	e biosynthetic process				5287917							
GOTERM_BP_FAT	GO:0051186~cofactor metabolic process	8	8	0.056258	5261315, 5274886, 5286049, 5277618, 5284767, 5297155, 5288791, 5287917	85	684	16709	2.29914	1	0.461721	54.74787
GOTERM_BP_FAT	GO:0042559~pteridine and derivative biosynthetic process	3	3	0.061341	5261315, 5274886, 5286049	85	80	16709	7.371618	1	0.471891	57.97377
GOTERM_BP_FAT	GO:0042558~pteridine and derivative metabolic process	3	3	0.061341	5261315, 5274886, 5286049	85	80	16709	7.371618	1	0.471891	57.97377
GOTERM_BP_FAT	GO:0006732~coenzyme metabolic process	5	5	0.209177	5261315, 5274886, 5286049, 5297155, 5287917	85	468	16709	2.100176	1	0.790507	95.97947
Annotation Cluster 12	Enrichment Score: 1.47726869394448											
Category	Term	Count	%	PValue	Genes	List Total	Pop Hits	Pop Total	Fold Enrichment	Bonferroni	Benjamini	FDR
KEGG_PATHWAY	ecd00190:Oxidative phosphorylation	3	3	0.031923	5283429, 5300277, 5291861	51	40	7107	10.45147	1	0.580026	38.81659
KEGG_PATHWAY	ecw00190:Oxidative phosphorylation	3	3	0.033411	5283429, 5300277, 5291861	51	41	7107	10.19656	1	0.583663	40.22591
KEGG_PATHWAY	ecf00190:Oxidative phosphorylation	3	3	0.033411	5283429, 5300277, 5291861	51	41	7107	10.19656	1	0.583663	40.22591
KEGG_PATHWAY	ecm00190:Oxidative phosphorylation	3	3	0.033411	5283429, 5300277, 5291861	51	41	7107	10.19656	1	0.583663	40.22591
KEGG_PATHWAY	ect00190:Oxidative phosphorylation	3	3	0.033411	5283429, 5300277, 5291861	51	41	7107	10.19656	1	0.583663	40.22591
KEGG_PATHWAY	ece00190:Oxidative phosphorylation	3	3	0.033411	5283429, 5300277, 5291861	51	41	7107	10.19656	1	0.583663	40.22591
KEGG_PATHWAY	eck00190:Oxidative phosphorylation	3	3	0.033411	5283429, 5300277, 5291861	51	41	7107	10.19656	1	0.583663	40.22591
KEGG_PATHWAY	eum00190:Oxidative phosphorylation	3	3	0.033411	5283429, 5300277, 5291861	51	41	7107	10.19656	1	0.583663	40.22591
KEGG_PATHWAY	ecc00190:Oxidative phosphorylation	3	3	0.033411	5283429, 5300277, 5291861	51	41	7107	10.19656	1	0.583663	40.22591
KEGG_PATHWAY	ecr00190:Oxidative phosphorylation	3	3	0.033411	5283429, 5300277, 5291861	51	41	7107	10.19656	1	0.583663	40.22591
KEGG_PATHWAY	ecz00190:Oxidative phosphoryl	3	3	0.033411	5283429, 5300277, 5291861	51	41	7107	10.19656	1	0.583663	40.22591

	ation											
KEGG_PATHWAY	ecj00190:Oxidative phosphorylation	3	3	0.033411	5283429, 5300277, 5291861	51	41	7107	10.19656	1	0.583663	40.22591
KEGG_PATHWAY	ecg00190: Oxidative phosphorylation	3	3	0.033411	5283429, 5300277, 5291861	51	41	7107	10.19656	1	0.583663	40.22591
KEGG_PATHWAY	ecx00190: Oxidative phosphorylation	3	3	0.033411	5283429, 5300277, 5291861	51	41	7107	10.19656	1	0.583663	40.22591
KEGG_PATHWAY	ecq00190: Oxidative phosphorylation	3	3	0.033411	5283429, 5300277, 5291861	51	41	7107	10.19656	1	0.583663	40.22591
KEGG_PATHWAY	eci00190:Oxidative phosphorylation	3	3	0.033411	5283429, 5300277, 5291861	51	41	7107	10.19656	1	0.583663	40.22591
KEGG_PATHWAY	eco00190: Oxidative phosphorylation	3	3	0.033411	5283429, 5300277, 5291861	51	41	7107	10.19656	1	0.583663	40.22591
Annotation Cluster 13	Enrichment Score: 1.432379692690416											
Category	Term	Count	%	PValue	Genes	List Total	Pop Hits	Pop Total	Fold Enrichment	Bonferroni	Benjamini	FDR
SP_PIR_KEYWORDS	cell shape	3	3	0.010002	5304594, 5276311, 5307123	100	72	47487	19.78625	0.837882	0.040507	11.65594
GOTERM_BP_FAT	GO:0008360~regulation of cell shape	3	3	0.071022	5304594, 5276311, 5307123	85	87	16709	6.778499	1	0.486656	63.53617
GOTERM_BP_FAT	GO:0022604~regulation of cell morphogenesis	3	3	0.071022	5304594, 5276311, 5307123	85	87	16709	6.778499	1	0.486656	63.53617
Annotation Cluster 14	Enrichment Score: 1.370402820696135											
Category	Term	Count	%	PValue	Genes	List Total	Pop Hits	Pop Total	Fold Enrichment	Bonferroni	Benjamini	FDR
SP_PIR_KEYWORDS	electron transport	5	5	7.09E-04	5280453, 5279158, 5279860, 5275908, 5291861	100	191	47487	12.43115	0.120407	0.005815	0.870088
GOTERM_BP_FAT	GO:0006091~generation of precursor metabolites and energy	9	9	0.033696	5280453, 5283429, 5296877, 5279158, 5300277, 5279860, 5275908, 5291861, 5287917	85	746	16709	2.371566	0.999994	0.383676	37.46139
GOTERM_BP_FAT	GO:0009061~anaerobic respiration	4	4	0.046066	5279158, 5300277, 5275908, 5287917	85	159	16709	4.94532	1	0.425884	47.57705
GOTERM_BP_FAT	GO:0015980~energy derivation by oxidation of organic compounds	6	6	0.059399	5296877, 5279158, 5300277, 5275908, 5291861, 5287917	85	418	16709	2.821672	1	0.470475	56.76738
GOTERM_	GO:002290	5	5	0.0811	5280453, 5279158,	85	325	16709	3.0242	1	0.517	68.61

BP_FAT	0~electron transport chain			44	5279860, 5275908, 5291861				53		417	598
GOTERM_BP_FAT	GO:0045333~cellular respiration	5	5	0.116753	5279158, 5300277, 5275908, 5291861, 5287917	85	371	16709	2.649279	1	0.62239	81.73413
GOTERM_MF_FAT	GO:0009055~electron carrier activity	6	6	0.412615	5307481, 5279158, 5300277, 5279860, 5275908, 5291861	73	1039	17785	1.406911	1	0.9805	99.86174
Annotation Cluster 15	Enrichment Score: 1.335312403098097											
Category	Term	Count	%	PValue	Genes	List Total	Pop Hits	Pop Total	Fold Enrichment	Bonferroni	Benjamini	FDR
SP_PIR_KEYWORDS	pyridoxal phosphate	5	5	0.001523	5291640, 5286049, 5257800, 5301917, 5292331	100	235	47487	10.10362	0.241152	0.011432	1.862114
SP_PIR_KEYWORDS	lyase	6	6	0.024826	5285358, 5291640, 5286049, 5297155, 5301917, 5258016	100	788	47487	3.615761	0.989436	0.090443	26.65109
GOTERM_MF_FAT	GO:0019842~vitamin binding	6	6	0.049742	5274010, 5291640, 5286049, 5301917, 5292331, 5287917	73	494	17785	2.95907	0.99992	0.467017	46.81165
INTERPRO	IPR015421:Pyridoxal phosphate-dependent transferase, major region, subdomain 1	3	3	0.163498	5291640, 5301917, 5292331	96	271	35585	4.103436	1	1	90.52601
GOTERM_MF_FAT	GO:0030170~pyridoxal phosphate binding	4	4	0.17786	5291640, 5286049, 5301917, 5292331	73	359	17785	2.714542	1	0.85146	91.13749
GOTERM_MF_FAT	GO:0070279~vitamin B6 binding	4	4	0.17786	5291640, 5286049, 5301917, 5292331	73	359	17785	2.714542	1	0.85146	91.13749
Annotation Cluster 16	Enrichment Score: 1.2031618015943453											
Category	Term	Count	%	PValue	Genes	List Total	Pop Hits	Pop Total	Fold Enrichment	Bonferroni	Benjamini	FDR
GOTERM_MF_FAT	GO:0003723~RNA binding	7	7	0.006579	5285358, 5260574, 5301931, 5266128, 5277581, 5262183, 5258016	73	414	17785	4.11935	0.705132	0.126888	7.843544
GOTERM_BP_FAT	GO:0034660~ncRNA metabolic process	7	7	0.011783	5285358, 5287199, 5266128, 5276530, 5301917, 5271238, 5258016	85	377	16709	3.649961	0.984762	0.258334	14.98238
SP_PIR_KEYWORDS	lyase	6	6	0.024826	5285358, 5291640, 5286049, 5297155, 5301917, 5258016	100	788	47487	3.615761	0.989436	0.090443	26.65109
GOTERM_BP_FAT	GO:0034470~ncRNA processing	5	5	0.064481	5285358, 5266128, 5276530, 5301917, 5258016	85	300	16709	3.276275	1	0.470549	59.85883
GOTERM_BP_FAT	GO:0009451~RNA modification	4	4	0.072487	5285358, 5276530, 5301917, 5258016	85	192	16709	4.095343	1	0.485245	64.3157
GOTERM_BP_FAT	GO:0006396~RNA processing	5	5	0.110125	5285358, 5266128, 5276530, 5301917, 5258016	85	363	16709	2.707665	1	0.616269	79.76494
GOTERM_BP_FAT	GO:0006364~rRNA processing	3	3	0.110674	5285358, 5266128, 5258016	85	113	16709	5.218844	1	0.609761	79.93516

GOTERM_BP_FAT	GO:0016072~rRNA metabolic process	3	3	0.110674	5285358, 5266128, 5258016	85	113	16709	5.218844	1	0.609761	79.93516
GOTERM_BP_FAT	GO:0022613~ribonucleoprotein complex biogenesis	3	3	0.123831	5285358, 5266128, 5258016	85	121	16709	4.873797	1	0.637404	83.63981
GOTERM_BP_FAT	GO:0042254~ribosome biogenesis	3	3	0.123831	5285358, 5266128, 5258016	85	121	16709	4.873797	1	0.637404	83.63981
SP_PIR_KEYWORDS	Isomerase	3	3	0.313003	5285358, 5276530, 5258016	100	543	47487	2.623591	1	0.677784	99.02313
Annotation Cluster 17	Enrichment Score: 1.1350805223391942											
Category	Term	Count	%	PValue	Genes	List Total	Pop Hits	Pop Total	Fold Enrichment	Bonferroni	Benjamini	FDR
SP_PIR_KEYWORDS	iron	6	6	0.012095	5261315, 5307481, 5275125, 5279158, 5288791, 5275908	100	654	47487	4.356606	0.88947	0.047765	13.9311
SP_PIR_KEYWORDS	iron-sulfur	5	5	0.016948	5261315, 5307481, 5279158, 5288791, 5275908	100	469	47487	5.06258	0.954675	0.063708	19.00166
SP_PIR_KEYWORDS	4fe-4s	4	4	0.027071	5261315, 5279158, 5288791, 5275908	100	309	47487	6.147184	0.993039	0.096406	28.70573
GOTERM_MF_FAT	GO:0005506~iron ion binding	7	7	0.154244	5261315, 5307481, 5275125, 5279158, 5288791, 5275908, 5291861	73	896	17785	1.90336	1	0.855864	87.41812
GOTERM_MF_FAT	GO:0051536~iron-sulfur cluster binding	6	6	0.201716	5261315, 5307481, 5277618, 5279158, 5288791, 5275908	73	771	17785	1.895954	1	0.849606	93.84383
GOTERM_MF_FAT	GO:0051540~metal cluster binding	6	6	0.201716	5261315, 5307481, 5277618, 5279158, 5288791, 5275908	73	771	17785	1.895954	1	0.849606	93.84383
GOTERM_MF_FAT	GO:0051539~4 iron, 4 sulfur cluster binding	4	4	0.325486	5261315, 5279158, 5288791, 5275908	73	496	17785	1.964759	1	0.951938	99.23447
Annotation Cluster 18	Enrichment Score: 0.9540042717875992											
Category	Term	Count	%	PValue	Genes	List Total	Pop Hits	Pop Total	Fold Enrichment	Bonferroni	Benjamini	FDR
KEGG_PATHWAY	ect00230:Purine metabolism	3	3	0.099579	5279667, 5296304, 5301118	51	76	7107	5.500774	1	0.892195	79.57476
KEGG_PATHWAY	eum00230:Purine metabolism	3	3	0.104016	5279667, 5296304, 5301118	51	78	7107	5.359729	1	0.896242	81.04693
KEGG_PATHWAY	ecd00230:Purine metabolism	3	3	0.106255	5279667, 5296304, 5301118	51	79	7107	5.291884	1	0.894909	81.75158
KEGG_PATHWAY	ecz00230:Purine metabolism	3	3	0.106255	5279667, 5296304, 5301118	51	79	7107	5.291884	1	0.894909	81.75158
KEGG_PATHWAY	ecx00230:Purine metabolism	3	3	0.106255	5279667, 5296304, 5301118	51	79	7107	5.291884	1	0.894909	81.75158

KEGG_PATHWAY	ecg00230: Purine metabolism	3	3	0.106255	5279667, 5296304, 5301118	51	79	7107	5.291884	1	0.894909	81.75158
KEGG_PATHWAY	ecq00230: Purine metabolism	3	3	0.108507	5279667, 5296304, 5301118	51	80	7107	5.225735	1	0.893677	82.43565
KEGG_PATHWAY	ecr00230:Purine metabolism	3	3	0.110772	5279667, 5296304, 5301118	51	81	7107	5.16122	1	0.892539	83.09943
KEGG_PATHWAY	ecw00230: Purine metabolism	3	3	0.110772	5279667, 5296304, 5301118	51	81	7107	5.16122	1	0.892539	83.09943
KEGG_PATHWAY	eck00230: Purine metabolism	3	3	0.11305	5279667, 5296304, 5301118	51	82	7107	5.098278	1	0.89149	83.74323
KEGG_PATHWAY	ecc00230: Purine metabolism	3	3	0.11305	5279667, 5296304, 5301118	51	82	7107	5.098278	1	0.89149	83.74323
KEGG_PATHWAY	ecv00230: Purine metabolism	3	3	0.11534	5279667, 5296304, 5301118	51	83	7107	5.036853	1	0.890525	84.36737
KEGG_PATHWAY	ecm00230: Purine metabolism	3	3	0.11534	5279667, 5296304, 5301118	51	83	7107	5.036853	1	0.890525	84.36737
KEGG_PATHWAY	ecj00230:Purine metabolism	3	3	0.11534	5279667, 5296304, 5301118	51	83	7107	5.036853	1	0.890525	84.36737
KEGG_PATHWAY	eco00230: Purine metabolism	3	3	0.11534	5279667, 5296304, 5301118	51	83	7107	5.036853	1	0.890525	84.36737
KEGG_PATHWAY	ecf00230:Purine metabolism	3	3	0.117642	5279667, 5296304, 5301118	51	84	7107	4.976891	1	0.889638	84.9722
KEGG_PATHWAY	eci00230:Purine metabolism	3	3	0.117642	5279667, 5296304, 5301118	51	84	7107	4.976891	1	0.889638	84.9722
KEGG_PATHWAY	ece00230: Purine metabolism	3	3	0.122282	5279667, 5296304, 5301118	51	86	7107	4.861149	1	0.893769	86.12528
Annotation Cluster 19	Enrichment Score: 0.9202577075926217											
Category	Term	Count	%	PValue	Genes	List Total	Pop Hits	Pop Total	Fold Enrichment	Bonferroni	Benjamini	FDR
GOTERM_BP_FAT	GO:0008610~lipid biosynthetic process	7	7	0.03933	5302495, 5260139, 5286820, 5278574, 5297155, 5274212, 5292331	85	498	16709	2.763123	0.999999	0.39701	42.2744
GOTERM_BP_FAT	GO:0008654~phospholipid biosynthetic process	3	3	0.156424	5286820, 5274212, 5292331	85	140	16709	4.212353	1	0.706376	90.26511
GOTERM_BP_FAT	GO:0006644~phospholipid metabolic process	3	3	0.181372	5286820, 5274212, 5292331	85	154	16709	3.829412	1	0.756561	93.54653
GOTERM_	GO:001963	3	3	0.1867	5286820, 5274212,	85	157	16709	3.7562	1	0.760	94.10

BP_FAT	7~organophosphate metabolic process			97	5292331				38		978	81
Annotation Cluster 20	Enrichment Score: 0.8560055669734629											
Category	Term	Count	%	PValue	Genes	List Total	Pop Hits	Pop Total	Fold Enrichment	Bonferroni	Benjamini	FDR
GOTERM_BP_FAT	GO:0034654~nucleobase, nucleoside, nucleotide and nucleic acid biosynthetic process	5	5	0.084711	5283429, 5276530, 5297155, 5296304, 5279860	85	330	16709	2.978431	1	0.524767	70.24408
GOTERM_BP_FAT	GO:0034404~nucleobase, nucleoside and nucleotide biosynthetic process	5	5	0.084711	5283429, 5276530, 5297155, 5296304, 5279860	85	330	16709	2.978431	1	0.524767	70.24408
GOTERM_BP_FAT	GO:0009165~nucleotide biosynthetic process	3	3	0.376794	5283429, 5297155, 5279860	85	260	16709	2.26819	1	0.946524	99.84595
Annotation Cluster 21	Enrichment Score: 0.6208475735013312											
Category	Term	Count	%	PValue	Genes	List Total	Pop Hits	Pop Total	Fold Enrichment	Bonferroni	Benjamini	FDR
SP_PIR_KEYWORDS	protein transport	3	3	0.014977	5296702, 5257606, 5274831	100	89	47487	16.00685	0.934866	0.057648	16.97631
GOTERM_MF_FAT	GO:0008565~protein transporter activity	4	4	0.160041	5296702, 5269304, 5302970, 5274831	73	341	17785	2.857832	1	0.850119	88.44474
GOTERM_BP_FAT	GO:0015031~protein transport	5	5	0.280024	5296702, 5269304, 5302970, 5257606, 5274831	85	533	16709	1.844057	1	0.878594	98.88804
GOTERM_BP_FAT	GO:0045184~establishment of protein localization	5	5	0.280024	5296702, 5269304, 5302970, 5257606, 5274831	85	533	16709	1.844057	1	0.878594	98.88804
GOTERM_BP_FAT	GO:0008104~protein localization	5	5	0.290166	5296702, 5269304, 5302970, 5257606, 5274831	85	542	16709	1.813436	1	0.884719	99.08436
GOTERM_BP_FAT	GO:0009306~protein secretion	3	3	0.582787	5296702, 5269304, 5302970	85	387	16709	1.523849	1	0.994647	99.99937
GOTERM_BP_FAT	GO:0032940~secretion by cell	3	3	0.582787	5296702, 5269304, 5302970	85	387	16709	1.523849	1	0.994647	99.99937
GOTERM_BP_FAT	GO:0046903~secretion	3	3	0.582787	5296702, 5269304, 5302970	85	387	16709	1.523849	1	0.994647	99.99937
Annotation Cluster 22	Enrichment Score: 0.5231762555810396											
Category	Term	Count	%	PValue	Genes	List Total	Pop Hits	Pop Total	Fold Enrich	Bonferroni	Benjamini	FDR

									ment			
SP_PIR_KEYWORDS	ATP	4	4	0.005498	5270689, 5287199, 5271238, 5275617	100	170	47487	11.17341	0.631309	0.026607	6.570698
SP_PIR_KEYWORDS	nucleotide-binding	9	9	0.037633	5261315, 5270689, 5273276, 5283477, 5302805, 5287199, 5297155, 5271238, 5275617	100	1831	47487	2.334151	0.999035	0.129652	37.68277
SP_PIR_KEYWORDS	atp-binding	7	7	0.16174	5270689, 5283477, 5302805, 5287199, 5297155, 5271238, 5275617	100	1760	47487	1.888688	1	0.446424	88.64083
GOTERM_MF_FAT	GO:0000166~nucleotide binding	16	16	0.297456	5270689, 5273276, 5302805, 5283477, 5297155, 5304594, 5271238, 5268716, 5307123, 5275617, 5308498, 5261315, 5271346, 5287199, 5284767, 5300277	73	3175	17785	1.227742	1	0.941558	98.73303
GOTERM_MF_FAT	GO:0017076~purine nucleotide binding	12	12	0.529086	5261315, 5270689, 5271346, 5273276, 5283477, 5302805, 5287199, 5297155, 5304594, 5271238, 5307123, 5275617	73	2677	17785	1.092104	1	0.995292	99.99103
GOTERM_MF_FAT	GO:0032555~purine ribonucleotide binding	10	10	0.641938	5261315, 5270689, 5273276, 5283477, 5302805, 5287199, 5297155, 5271238, 5307123, 5275617	73	2385	17785	1.02151	1	0.999121	99.9997
GOTERM_MF_FAT	GO:0032553~ribonucleotide binding	10	10	0.641938	5261315, 5270689, 5273276, 5283477, 5302805, 5287199, 5297155, 5271238, 5307123, 5275617	73	2385	17785	1.02151	1	0.999121	99.9997
GOTERM_MF_FAT	GO:0030554~adenyl nucleotide binding	10	10	0.70961	5270689, 5271346, 5283477, 5302805, 5287199, 5297155, 5304594, 5271238, 5307123, 5275617	73	2523	17785	0.965637	1	0.999717	99.99998
GOTERM_MF_FAT	GO:0001883~purine nucleoside binding	10	10	0.70961	5270689, 5271346, 5283477, 5302805, 5287199, 5297155, 5304594, 5271238, 5307123, 5275617	73	2523	17785	0.965637	1	0.999717	99.99998
GOTERM_MF_FAT	GO:0001882~nucleoside binding	10	10	0.728577	5270689, 5271346, 5283477, 5302805, 5287199, 5297155, 5304594, 5271238, 5307123, 5275617	73	2565	17785	0.949825	1	0.999756	99.99999
GOTERM_MF_FAT	GO:0005524~ATP binding	8	8	0.812534	5270689, 5283477, 5302805, 5287199, 5297155, 5271238, 5307123, 5275617	73	2225	17785	0.875974	1	0.999937	100
GOTERM_MF_FAT	GO:0032559~adenyl ribonucleotide binding	8	8	0.814774	5270689, 5283477, 5302805, 5287199, 5297155, 5271238, 5307123, 5275617	73	2231	17785	0.873618	1	0.999922	100
Annotation Cluster 23	Enrichment Score: 0.280022572880297											
Category	Term	Count	%	PValue	Genes	List Total	Pop Hits	Pop Total	Fold Enrichment	Bonferroni	Benjamini	FDR
SP_PIR_KEYWORDS	atp-binding	7	7	0.16174	5270689, 5283477, 5302805, 5287199, 5297155, 5271238, 5275617	100	1760	47487	1.888688	1	0.446424	88.64083
INTERPRO	IPR017871:ABC transporter	3	3	0.352957	5270689, 5297155, 5275617	96	465	35585	2.391465	1	1	99.68066

	, conserved site											
INTERPRO	IPR003439:ABC transporter-like	3	3	0.410849	5270689, 5297155, 5275617	96	526	35585	2.114128	1	1	99.90734
SMART	SM00382:AAA	3	3	0.629486	5270689, 5297155, 5275617	13	852	5022	1.360238	0.999999	0.999999	99.88226
INTERPRO	IPR003593:ATPase, AAA+ type, core	3	3	0.66724	5270689, 5297155, 5275617	96	852	35585	1.305201	1	1	99.99995
GOTERM_MF_FAT	GO:0005524~ATP binding	8	8	0.812534	5270689, 5283477, 5302805, 5287199, 5297155, 5271238, 5307123, 5275617	73	2225	17785	0.875974	1	0.999937	100
GOTERM_MF_FAT	GO:0032559~adenyl ribonucleotide binding	8	8	0.814774	5270689, 5283477, 5302805, 5287199, 5297155, 5271238, 5307123, 5275617	73	2231	17785	0.873618	1	0.999922	100
GOTERM_MF_FAT	GO:0016887~ATPase activity	3	3	0.881966	5270689, 5297155, 5275617	73	891	17785	0.820303	1	0.999991	100
Annotation Cluster 24	Enrichment Score: 0.20143654986348433											
Category	Term	Count	%	PValue	Genes	List Total	Pop Hits	Pop Total	Fold Enrichment	Bonferroni	Benjamini	FDR
SP_PIR_KEYWORDS	dna-binding	8	8	0.173302	5283993, 5271647, 5307240, 5256824, 5283477, 5300514, 5266110, 5280283	100	2192	47487	1.733102	1	0.459429	90.42856
SP_PIR_KEYWORDS	Transcription	7	7	0.194341	5283993, 5256824, 5283477, 5300514, 5266110, 5279667, 5280283	100	1866	47487	1.781399	1	0.49052	93.03451
SP_PIR_KEYWORDS	transcription regulation	6	6	0.346742	5283993, 5256824, 5283477, 5300514, 5266110, 5280283	100	1860	47487	1.531839	1	0.717305	99.47495
INTERPRO	IPR011991:Winged helix repressor DNA-binding	4	4	0.499474	5283993, 5300514, 5266110, 5280283	96	997	35585	1.48717	1	1	99.98923
UP_SEQ_FEATURE	DNA-binding region:H-T-H motif	5	5	0.666058	5283993, 5256824, 5300514, 5266110, 5280283	100	431	9468	1.098376	1	1	99.99989
GOTERM_MF_FAT	GO:0043565~sequence-specific DNA binding	3	3	0.777911	5283993, 5300514, 5266110	73	695	17785	1.051641	1	0.999907	100
GOTERM_MF_FAT	GO:0003700~transcription factor activity	5	5	0.921452	5283993, 5256824, 5300514, 5266110, 5280283	73	1694	17785	0.719097	1	0.999999	100
GOTERM_BP_FAT	GO:0006350~transcription	7	7	0.92361	5283993, 5256824, 5283477, 5300514, 5266110, 5279667, 5280283	85	1886	16709	0.729605	1	1	100
GOTERM_MF_FAT	GO:0030528~transcription regulator activity	6	6	0.940901	5283993, 5256824, 5283477, 5300514, 5266110, 5280283	73	2118	17785	0.69017	1	1	100
GOTERM_BP_FAT	GO:0045449~regulation of	6	6	0.997963	5283993, 5256824, 5283477, 5300514, 5266110, 5280283	85	2591	16709	0.455214	1	1	100

	transcription											
GOTERM_BP_FAT	GO:0006355~regulation of transcription, DNA-dependent	5	5	0.998185	5283993, 5256824, 5300514, 5266110, 5280283	85	2311	16709	0.425306	1	1	100
GOTERM_BP_FAT	GO:0051252~regulation of RNA metabolic process	5	5	0.998233	5283993, 5256824, 5300514, 5266110, 5280283	85	2317	16709	0.424205	1	1	100
GOTERM_MF_FAT	GO:0003677~DNA binding	10	10	0.999844	5283993, 5271647, 5264271, 5307240, 5256824, 5283477, 5300514, 5266110, 5279667, 5280283	73	5283	17785	0.461159	1	1	100
